# P2X4 purinergic receptor loss alters macrophage homeostasis, cytokine secretion, and phagocytosis in a myeloid-specific P2X4 purinergic receptor knockout mouse

**DOI:** 10.3389/fimmu.2026.1916582

**Published:** 2026-09-10

**Authors:** Vahinipriya Manoharan, Kennedy M. Rains, Tristan R. Ibarra, Ashwin Barath Vaidhyalingham, Taylor R. Pistone, Praise O. Awogbesan, Fernanda Caramella, Caylee D. Kelso, Jelani O. Jarrett, Melanie Quiñones Llanos, Sophia O. Olatunji-Richard, Rieko Muramatsu, David E. Sanin, Janielle P. Maynard

**Affiliations:** 1 Department of Pathology, Johns Hopkins University School of Medicine, Baltimore, MD, United States; 2 Department of Urology, James Buchanan Brady Urological Institute, Johns Hopkins University School of Medicine, Baltimore, MD, United States; 3 Bloomberg~Kimmel Institute for Cancer Immunotherapy, Johns Hopkins University School of Medicine, Baltimore, MD, United States; 4 Department of Molecular Pharmacology, National Institute of Neuroscience, National Center of Neurology and Psychiatry, Tokyo, Japan; 5 Sidney Kimmel Comprehensive Cancer Center, Johns Hopkins University School of Medicine, Baltimore, MD, United States

**Keywords:** angiogenesis, cytokine secretion, LysMcre mice, macrophages, P2X4 purinergic receptor, purinergic signaling, tissue-resident macrophages, phagocytosis

## Abstract

**Introduction:**

The P2X4 purinergic receptor is robustly expressed on tumor-associated macrophages; however, the role of P2X4 purinergic receptors on macrophages is not fully defined. To investigate the functional role of P2X4 purinergic receptor in macrophages, we generated and characterized a myeloid-specific P2X4 purinergic receptor knockout (P2X4^ΔM^) mouse.

**Methods:**

Immune profiles were assessed across multiple tissues using multiplex immunohistochemistry and flow cytometry. Bulk RNA sequencing, gene set enrichment analysis (GSEA), and Gene Ontology over-representation analysis (ORA) were performed to determine the transcriptional consequence of P2X4 purinergic receptor loss in macrophages. Array and ELISA analyses of cytokine secretion, phagocytosis assays and Seahorse extracellular flux analysis of oxygen consumption were used to assess the functional role of P2X4 purinergic receptors in bone marrow-derived macrophages.

**Results:**

P2X4 purinergic receptor loss in macrophages from male mice resulted in reduced tissue-resident macrophage density in liver, spleen, and peritoneal tissues. In addition, male P2X4^ΔM^ mice had increased B cell populations among peritoneal cells. Bulk RNA sequencing analysis identified P2X4 purinergic receptor as the predominant P2 purinergic receptor expressed in murine macrophages and demonstrated that its deletion did not appear to induce compensatory expression changes in other P2 purinergic receptors. GSEA and Gene Ontology ORA determined an enrichment of interferon-β response and angiogenesis-related pathways in macrophages from male mice. Mitochondrial and metabolic pathways were enriched in macrophages from female mice. P2X4 purinergic receptor deficient macrophages exhibited reduced expression of pro-angiogenic Lysyl Oxidase-Like 2 whereas anti-angiogenic gene Plexin-D1 expression was increased. Functionally, P2X4 purinergic receptor deletion attenuated LPS-induced IL-6 and MCP-1 secretion in bone marrow derived macrophages and P2X4^ΔM^ macrophages exhibited increased phagocytic activity.

**Conclusions:**

Collectively, these findings demonstrate that macrophage specific P2X4 purinergic receptor signaling regulates macrophage homeostasis, cytokine secretion, phagocytosis, and alters the expression of angiogenesis-associated genes.

## 1 Introduction

The P2 purinergic receptor family comprises of P2X ligand-gated ion channel receptors (P2X1–P2X7) and P2Y G protein–coupled receptors (P2Y1, P2Y2, P2Y6, P2Y12–14) which are activated by extracellular nucleotides (1–3). Cellular stress results in ATP or UTP release into the extracellular milieu (4) where they and their derivatives activate P2 purinergic receptors to elicit diverse signaling pathways across the nervous, digestive, and immune systems (1, 5). In some instances, chronic activation and thereby receptor dysregulation contribute to disease development (5–8). Importantly, increased extracellular ATP concentrations act as a danger signal, recruiting immune cells and activating the inflammasome (9).

Among the immune cells recruited are macrophages, which express P2 purinergic receptors including P2X4, P2X7, and P2Y12 purinergic receptors (10). We previously reported P2X4 purinergic receptor expression on macrophages within the prostate tumor microenvironment (TME) (11). While P2 purinergic signaling has been implicated in regulating macrophage activation (12), cytokine production (13), and phagocytosis (14), the specific role of the P2X4 purinergic receptor in macrophage biology is not fully defined.

Macrophages are central regulators of tissue homeostasis and immune responses, contributing to host defense, tissue remodeling, angiogenesis, and metabolic regulation across diverse physiological and pathological contexts (15–17). Macrophages may be derived from circulating monocytes, but many are key components of tissue-resident immune populations, which actively shape local microenvironments within organs, including the TME (18–20).

To characterize P2X4 purinergic receptor function in macrophages using a model that recapitulates the physiological microenvironment during pathogenesis, we generated a myeloid-specific P2X4 purinergic receptor knockout (P2X4^ΔM^) mouse using the Cre-Lox recombination system with LysMcre transgenic mice. This approach enables targeted deletion of P2X4 purinergic receptor predominantly in macrophages and provides a platform to investigate its role across physiological and disease contexts.

Herein, we report efficient and macrophage-specific deletion of the P2X4 purinergic receptor in the P2X4^ΔM^ mouse. We show that P2X4 purinergic receptor loss in macrophages from male mice reduced tissue-resident macrophage abundance in the liver, spleen, and peritoneal cavity. In addition, P2X4 purinergic receptor deletion in murine macrophages results in significant transcriptional changes. Inflammatory and angiogenic pathways were enriched in macrophages from P2X4^ΔM^ male mice while metabolic pathways were enriched in macrophages from P2X4^ΔM^ female mice. Functionally, P2X4 purinergic receptor deletion attenuated lipopolysaccharide (LPS) induced macrophage IL-6 and MCP-1 secretion without altering IL-10 secretion. Our findings establish the P2X4^ΔM^ mouse as an appropriate model to study macrophage-specific P2X4 purinergic receptor function across different physiological contexts.

## 2 Materials and methods

### 2.1 Mice

Mice were housed under pathogen-free conditions on a 12-hour (h) light/dark cycle and received standard food and water ad libitum. All procedures were performed under the guidelines of Johns Hopkins University Animal Care and Use Committee (JHU ACUC). Mice were weaned 21 days from their birth. The litter size was defined as the number of live pups counted at the time of weaning. Animals were sacrificed by CO_2_ asphyxiation. Blood was collected by cardiac puncture for serum isolation. Liver, spleen, and brain tissues were formalin-fixed and paraffin-embedded (FFPE) for histological analyses.

### 2.2 Peritoneal lavage

Peritoneal cavity cells were collected by peritoneal lavage. Briefly, 8–10 mL of ice-cold PBS containing 2% FBS was injected into the peritoneal cavity, gently massaged, and then recovered. Cells were centrifuged at 400 × g for 4 minutes (min) at 4 °C, and the supernatant was removed. Red blood cells were lysed using Ammonium-Chloride-Potassium (ACK) lysis buffer (Cat. No. 118-156-101, Quality Biological, Gaithersburg, MD, USA) for 5 min at room temperature.

### 2.3 Bone marrow–derived macrophage preparation

Bone marrow was isolated from the femurs and tibias of mice. Briefly, bones were flushed with sterile phosphate-buffered saline (PBS) using a 25-gauge needle, and the resulting cell suspension was passed through a 70-µm cell strainer to remove debris. Cells were centrifuged at 400 × g for 4 min, resuspended in ACK cell lysis buffer, and incubated for 1 min at room temperature. Lysis was quenched by the addition of excess PBS, followed by centrifugation at 400 × g for 4 min. The cell pellet was resuspended in complete macrophage differentiation medium consisting of RPMI-1640 supplemented with 10% fetal bovine serum (FBS), 1% penicillin–streptomycin, and 20 ng/mL recombinant mouse macrophage colony-stimulating factor (M-CSF; Cat. No. 315-02, PeproTech, Rocky Hill, NJ, USA).

Cells were seeded in non-tissue-culture-treated Petri dishes and cultured at 37 °C in a humidified incubator with 5% CO_2_. Fresh M-CSF was added on days 2 and 5 of culture. Fully differentiated BMDMs were detached using PBS containing 2mM EDTA and harvested by gentle scraping for downstream experiments. Cell viability was assessed by trypan blue exclusion, and macrophage purity was confirmed by immunohistochemistry (IHC) analysis of F4/80.

### 2.4 Flow cytometry

Peritoneal cavity cells were resuspended in FACS buffer (PBS containing 2% FBS) containing Aqua viability dye (Cat. No. L34957, Thermo Fisher Scientific, Waltham, MA, USA) to exclude dead cells (**Supplementary Table 1**). Cells were washed with FACS buffer and incubated simultaneously with Fc block - anti-mouse CD16/CD32 (Cat. No. 101302, BioLegend, San Diego, CA, USA) to prevent non-specific binding and fluorochrome-conjugated antibodies against surface markers for 30 min at 4 °C in the dark. Cells were then washed with FACS buffer. For intracellular staining, cells were fixed and permeabilized using the fixation/permeabilization kit (Cat. No. 00-5521-00, eBioscience, San Diego, CA, USA) according to the manufacturer’s instructions. Intracellular markers were stained either with directly conjugated antibodies or using a primary antibody followed by a fluorochrome-conjugated secondary antibody (**Supplementary Table 1**). Data acquisition was performed on BD Fortessa (SKCCC Flow Cytometry Technology Development Center (FCTDC)) and analyzed using FlowJo software (version 10.10.0, BD). Single cells were gated based on forward and side scatter, and live cells were selected by excluding dead cells stained with viability dye. Compensation controls and fluorescence minus one (FMO) controls were used to set gating thresholds.

### 2.5 Fluorescence-activated cell sorting

Red blood cells were lysed from peritoneal cavity cells using ACK cell lysis buffer, and cells were stained with Aqua viability dye. Cells were washed with FACS buffer and incubated simultaneously with Fc block (anti-mouse CD16/CD32, clone 93; Cat. No. 101302, BioLegend, San Diego, CA, USA) and fluorochrome-conjugated antibodies against surface markers (CD11b, F4/80) for 30 min at 4 °C in the dark (**Supplementary Table 1**). After staining, cells were washed with FACS buffer and resuspended in RPMI supplemented with 2% FBS. Data acquisition and sorting of live, singlet, CD11b^+^, F4/80^+^ peritoneal macrophages were performed on a BSL-1^+^ Fusion Sorter using BD FACSDiva™ Software at the SKCCC Flow Cytometry Technology Development Center (FCTDC). Sorted macrophages were either used to prepare the cytospin slides or directly collected into TRIzol for RNA extraction.

### 2.6 Immunohistochemistry

IHC was performed on formalin fixed cytospin slides of sorted peritoneal macrophages or BMDMs. Slides were baked at 60 °C and rehydrated through graded ethanol to distilled water. Heat-induced antigen retrieval was performed using optimized retrieval buffers. Endogenous peroxidase activity was quenched using Dual Endogenous Enzyme-Blocking Reagent (Cat. No. S2003, Agilent Technologies, Santa Clara, CA, USA). Slides were incubated with primary antibodies against target proteins, rabbit polyclonal anti-P2X4 (Cat. No. APR-002, Alomone Labs, Jerusalem, Israel) and rabbit monoclonal anti-F4/80 (Cat. No. 70076S, Cell Signaling Technology, Danvers, MA, USA), followed by incubation with PowerVision Poly-HRP Anti-Rabbit IgG secondary antibodies (Cat. No. PV6119, Leica Biosystems, Deer Park, IL, USA). Chromogenic detection was performed using 3,3′-diaminobenzidine (DAB; Cat. No. D4293, MilliporeSigma, Burlington, MA, USA). Slides were counterstained with hematoxylin, dehydrated through graded ethanol, cleared in xylene, and mounted using Cytoseal™ mounting medium (Cat. No. 8310-4, Epredia, Kalamazoo, MI, USA). Slides were scanned with a ×40 objective using Ventana DP200 (Roche Diagnostics) digital whole slide scanner.

### 2.7 Multiplex IHC

mIHC was performed on FFPE mouse tissue sections using sequential chromogenic detection with 3-amino-9-ethylcarbazole (AEC) as previously described (21). Briefly, sections were baked at 60 °C for 10 min, followed by deparaffinization in xylene and rehydration through graded ethanol to distilled water. Heat-induced antigen retrieval was performed using optimized retrieval buffers and microwave heating. Endogenous peroxidase activity was quenched using Dual Endogenous Enzyme-Blocking Reagent.

Liver and spleen tissues were sequentially stained with primary antibodies against rabbit polyclonal anti-P2X4 (Cat. No. APR-002, Alomone Labs, Jerusalem, Israel), rabbit monoclonal anti-CD3 (Cat. No. RM9107S0, Epredia, Kalamazoo, MI, USA), and rabbit monoclonal anti-F4/80 (Cat. No. 70076S, Cell Signaling Technology, Danvers, MA, USA; **Supplementary Figure 1A**).

Brain tissues were sequentially stained using primary antibodies against rabbit polyclonal anti- P2X4 (Cat. No. APR-002, Alomone Labs, Jerusalem, Israel), rabbit monoclonal anti-Iba1 (Cat. No. ab178846, Abcam, Cambridge, UK), and rabbit monoclonal anti-P2Y12 (Cat. No. ab300140, Abcam, Cambridge, UK; **Supplementary Figure 1B**).

Following primary antibody incubation, sections were incubated with PowerVision Poly-HRP Anti-Rabbit IgG secondary antibodies (Cat. No. PV6119, Leica Biosystems, Deer Park, IL, USA), and chromogenic signal was developed using AEC substrate (Cat. No. SK-4205, Vector Laboratories, Burlingame, CA, USA. Slides were counterstained with hematoxylin, mounted using an aqueous mounting medium (Cat. No. ZK1025, Vector Laboratories, Burlingame, CA, USA), and slides were scanned with a ×40 objective using Ventana DP200 (Roche Diagnostics) digital whole slide scanner.

After image acquisition, AEC chromogen was removed using an alcohol-based stripping method, followed by additional antigen retrieval or SDS-based primary antibody stripping, as optimized for each antibody. Complete removal of prior staining was confirmed by performing IHC without the primary antibody (**Supplementary Figure 1**). This sequential staining, imaging, and stripping process was repeated for each antibody included in the multiplex panel (**Supplementary Figure 1**).

Quantitative image analysis was performed using HALO image analysis software (HALO v3.6.4134.396, Indica Labs). Tissue regions of interest (ROIs) were manually annotated to include relevant tissue and exclude artifacts, folds, and non-tissue areas. Within each staining batch, thresholds and algorithm parameters were applied consistently across all samples and experimental groups. Single-marker expression was quantified by measuring the percentage of AEC-positive area within the annotated ROIs. For dual-marker analysis of P2X4 and F4/80, images from sequential staining rounds were aligned using the HALO Tissue Registration module to generate fused images, and the percentage of overlapping AEC-positive area representing dual P2X4/F4/80 expression was quantified within the annotated ROIs (**Supplementary Figure 2**).

### 2.8 Bulk RNA sequencing

RNA extraction, library preparation and RNA sequencing were performed by the SKCCC Experimental and Computational Genomics Core facility at Johns Hopkins School of Medicine. Total RNA was extracted from sorted peritoneal macrophages using the AllPrep DNA/RNA Mini Kit (Cat. No. 80204, Qiagen, Hilden, Germany) according to the manufacturer’s instructions. RNA concentration was quantified using a NanoDrop spectrophotometer, and RNA quality was assessed using the Agilent 2100 Bioanalyzer. Three replicates per experimental condition were used.

For library preparation, 500 ng of total RNA per sample was used to generate stranded mRNA libraries using the TruSeq Stranded Total RNA Library Preparation Kit (Illumina), following the manufacturer’s protocol. Libraries were quantified using the Qubit fluorometer (Thermo Fisher Scientific) and sequenced on an Illumina NovaSeq 6000 X platform using 150 bp paired-end, dual-indexed reads, with 1% PhiX control spike-in. Sequencing depth was targeted at approximately 50 million reads per library.

Raw sequencing reads were subjected to adapter trimming, index demultiplexing, and standard quality control metrics. Processed reads were aligned to the mouse reference genome (mm39) using RSEM v1.3.3 with STAR alignment enabled. Gene expression quantification was performed using the rsem-calculate-expression module with the following parameters: --star, --calc-ci, --star-output-genome-bam, and --strandedness reverse.

Differential gene expression analysis and statistical testing were performed using DESeq2 v1.14.1. Intragroup variability and sample clustering were assessed by principal component analysis.

### 2.9 Gene set enrichment analysis

GSEA was performed using WebGestalt 2019 (https://2019.webgestalt.org/). All genes from the DESeq2 differential expression analysis were ranked according to the DESeq2 Wald test statistic and uploaded to WebGestalt, where they were mapped to Mus musculus Entrez Gene IDs. Enrichment analysis was conducted against GO, pathway, phenotype, and chromosomal location databases. The parameters were set as follows: minimum category size = 5, maximum category size = 2000, and multiple testing correction was performed using the Benjamini–Hochberg procedure false discovery rate (FDR) method. Gene sets with an FDR < 0.05 were considered significantly enriched.

### 2.10 Over-representation enrichment analysis

ORA enrichment analysis was performed using WebGestalt 2019 (WEB-based GEne SeT AnaLysis Toolkit, https://2019.webgestalt.org/). Significantly differentially expressed genes were uploaded and mapped to Mus musculus Entrez Gene IDs. The analysis was conducted against the GO Biological Process (non-redundant) database, with all protein-coding mouse genes used as the reference background. Parameters were set as follows: minimum category size = 5, maximum category size = 2000, and the Benjamini–Hochberg false discovery rate (FDR) method was applied for multiple testing correction. GO terms with FDR < 0.05 were considered significantly enriched.

### 2.11 Quantitative reverse transcriptase PCR

Total RNA was extracted from BMDMs using TRIzol (Cat. No. 15596026, Invitrogen, Carlsbad, CA, USA) according to the manufacturer’s instructions. RNA concentration and purity were assessed using a Multiskan SkyHigh Microplate Spectrophotometer with μDrop plate (Thermo Scientific™, Waltham, MA, USA). Complementary DNA (cDNA) was synthesized from 1 µg of total RNA using the SuperScript^®^ III First-Strand Synthesis System for RT-PCR (Cat. No. 18080-051, Invitrogen, Carlsbad, CA, USA) following the manufacturer’s protocol. Quantitative real-time PCR was performed using SYBR Green Master Mix (Cat. No. 170-8880, Bio-Rad Laboratories, Hercules, CA, USA) on a CFX Opus 96 Real-Time PCR instrument (Bio-Rad). Primers used for amplification are listed in **Supplementary Table 2**. Each reaction was performed in duplicate in a total volume of 20 µL. The cycling conditions were initial denaturation at 95 °C for 3 min, followed by 29 cycles of 95 °C for 15 seconds (sec) and annealing at primer-specific optimized temperatures for 30 sec (**Supplementary Table 2**). Relative gene expression levels were calculated using the 2^-ΔΔCt^ method and normalized to GAPDH.

### 2.12 Western blotting

Total protein extracts were prepared using RIPA lysis and extraction buffer (Cat. No. 89901, Thermo Fisher Scientific, Waltham, MA, USA) supplemented with Halt protease and phosphatase inhibitor cocktail (Cat. No. 78840, Thermo Fisher Scientific, Waltham, MA, USA) at a 1:100 dilution. Lysates were incubated on ice for 30 min with intermittent vortexing and centrifuged at 14,000 × g for 20 min at 4 °C. Protein concentration was determined using a BSA Standard-Pre-Diluted Set (Cat. No. 23208, Thermo Fisher Scientific, Waltham, MA, USA) with Reagents A (Cat. No. 23228, Thermo Fisher Scientific, Waltham, MA, USA) and B (Cat. No. 23224, Thermo Fisher Scientific, Waltham, MA, USA) to ensure equal protein loading across samples. Proteins were separated by SDS-PAGE, transferred to membranes, and membranes probed with primary antibodies against Lysyl Oxidase-Like 2 (Loxl2; Cat. No. NBP2-75559, Novus Biologicals, Centennial, CO, USA), Plexin-D1 (Plxnd1; Cat. No. NBP1-33634, Novus Biologicals, Centennial, CO, USA), Oxidative Phosphorylation (OXPHOS) Antibody Cocktail (CI-NDUFB8; NADH dehydrogenase [ubiquinone] 1 beta subcomplex subunit 8, CII-SDHB; Succinate Dehydrogenase Complex Iron Sulfur Subunit B, CIV-MTCO1; cytochrome c oxidase subunit1, CIII-UQCRC2; Ubiquinol-Cytochrome c Reductase Core Protein 2, CV-ATP5A; ATP synthase F1 subunit alpha; Cat. No. ab110413, Abcam, Cambridge, UK), and Heat Shock Protein 60 (HSP 60; Cat. No. ab121655, Abcam, Cambridge, UK). β-actin (Cat. No. A5441, Sigma-Aldrich, St. Louis, MO, USA) was used as a loading control. The membranes were then incubated with secondary antibodies HRP-linked Anti-Rabbit (Cat. No. 7074S, Cell Signaling Technology, Inc., Danvers, MA, USA) and Goat Anti-Mouse Alexa Fluor Plus 800 (Cat. No. A32730, Thermo Fisher Scientific, Waltham, MA, USA). The blots were imaged using the iBright CL1500 Imaging System (Cat. No. A44240, Thermo Fisher Scientific, Waltham, MA, USA).

### 2.13 Seahorse extracellular flux analysis

Cellular bioenergetics were assessed using a Seahorse XF96 Extracellular Flux Analyzer (Agilent Technologies, Santa Clara, CA, USA). BMDM cells were seeded in XF96 cell culture microplates at a density of 100,000 cells per well with Seahorse XF assay medium consisting of bicarbonate-free RPMI 1640 supplemented with 25 mM glucose, 1 mM sodium pyruvate, and 2 mM L-glutamine (pH 7.4). Cells were incubated for 1 h at 37 °C in a non-CO_2_ incubator before analysis.

The Seahorse sensor cartridge was hydrated overnight according to the manufacturer’s instructions. Oxygen consumption rate (OCR) and Extracellular Acidification Rate (ECAR) measurements were obtained under basal conditions and following sequential injections of metabolic modulators. Oligomycin (10 μM), Carbonyl cyanide p-trifluoromethoxyphenylhydrazone (FCCP; 10 μM), and a mixture of antimycin A (10 μM) and rotenone (1 μM) were loaded into the Seahorse injection ports and injected sequentially during the assay to assess mitochondrial respiration parameters. Three measurement cycles were performed before the first injection to establish basal metabolic activity, followed by three measurement cycles after each injection. Each measurement cycle consisted of 3 min mixing and 3 min measurement. Background correction was performed using cell-free wells containing assay medium only. The resulting OCR values were baseline-corrected (100*Value/mean[baseline]) for each well prior to analysis.

### 2.14 Cytokine array

Whole blood was collected from mice via cardiac puncture, allowed to clot at room temperature, and centrifuged to isolate serum. Serum samples were analyzed using the Proteome Profiler Mouse XL Cytokine Array (Bio-Techne, Minneapolis, MN, USA) according to the manufacturer’s instructions.

### 2.15 Enzyme-linked immunosorbent assay

Whole blood was collected from mice by cardiac puncture, allowed to clot at room temperature, and centrifuged to isolate serum. Serum samples were analyzed for IL-10, MCP-1, and GDF15 cytokine levels using Quantikine ELISA kits (Bio-Techne, Minneapolis, MN, USA) according to the manufacturer’s instructions.

BMDMs were treated with 20 ng/ml LPS for 18 h. Conditioned media were collected and analyzed for mouse IL-10, IL-6, and CCL2/JE/MCP-1 levels using DuoSet ELISA kits (Bio-Techne, Minneapolis, MN, USA) according to the manufacturer’s instructions. Absorbance was measured using a Multiskan™ SkyHigh Microplate Spectrophotometer (Thermo Scientific). Corrected standard values were used to generate standard curves and interpolate sample concentrations using GraphPad Prism software.

### 2.16 Phagocytosis assay with fluorescence bioparticles

Phagocytic activity was assessed using the Vybrant™ Phagocytosis Assay Kit (Cat. No. V6694, Thermo Fisher Scientific, Waltham, MA, USA) according to the manufacturer’s instructions. BMDMs were seeded in black-walled, clear-bottom 96 well plates at a density of 50,000 cells per well and allowed to adhere overnight. The following day, the culture medium was removed, and the cells were incubated with fluorescein-labeled Escherichia coli K-12 Bioparticles suspended in HBSS buffer at 37 °C in a humidified incubator with 5% CO_2_ for 2 h to allow phagocytosis. Following incubation, the medium containing non-internalized particles was removed. Trypan blue solution provided in the kit was added for 1 min at room temperature to quench extracellular fluorescence coming from non-phagocytosed Bioparticles. Fluorescence was measured immediately using a microplate reader with excitation/emission wavelengths of 480/520 nm. Background fluorescence from wells containing particles without cells was subtracted from all measurements. Phagocytosis was expressed as a percentage relative to the control group after normalization.

### 2.17 Statistical analysis

Data are presented as mean ± standard deviation (SD). Statistical analyses were performed using GraphPad Prism (version 10.1.2). Comparisons between two groups were conducted using the unpaired T test or Mann–Whitney U test. For comparisons among three or more groups, one-way or two-way analysis of variance (ANOVA) followed by appropriate *post hoc* tests was performed. A p-value < 0.05 was considered statistically significant.

## 3 Results

### 3.1 Development of P2X4^ΔM^ mice

P2X4^ΔM^ mice were generated using a Cre–loxP recombination strategy to achieve myeloid cell–specific deletion of the P2X4 purinergic receptor gene. Mice harboring loxP sites flanking a critical region of exon 2 of the P2X4 purinergic receptor (P2X4^fl/fl^) were kindly provided by Rieko Muramatsu at National Center of Neurology and Psychiatry, Japan (22). These mice were crossed with LysMcre transgenic mice (B6.129P2-Lyz2^tm1(cre)Ifo/J^) obtained from The Jackson Laboratory to enable recombination predominantly in macrophages. The LysMcre mice facilitates deletion of the targeted gene in the myeloid cell lineage including monocytes, mature macrophages, and granulocytes (23). The F1 generation from P2X4^fl/fl^ mice and LysMcre mice was heterozygous for both the floxed P2X4 allele and Cre recombinase (**Supplementary Figure 3A**). Genomic DNA was isolated from ear punches and genotyping was performed by Transnetyx, Inc. (Cordova, TN, USA) to identify P2X4^ΔM^ (P2X4^fl/fl^; Lyz2^cre/cre^) mice from the F2 generation. F2 generation littermates lacking both the floxed allele and Cre recombinase were used as WT controls (P2X4^+/+^; Lyz2-Cre^-/-^) to ensure a comparable genetic background with P2X4^ΔM^ mice. In addition, to assess whether insertion of the loxP sites altered basal P2X4 purinergic receptor expression, we compared WT and P2X4^fl/fl^; Lyz2-Cre^-/-^ mice by mIHC. P2X4 purinergic receptor protein expression in the F4/80^+^ macrophage population within the liver and spleen were comparable between the two groups (**Supplementary Figure 4** and **5**). P2X4^ΔM^ and WT mice were subsequently bred separately to maintain their respective colonies.

To confirm efficient and cell type–specific deletion of P2X4 purinergic receptors, peritoneal cavity macrophages, BMDMs, liver, spleen, and brain were harvested from P2X4^ΔM^ and WT mice. P2X4 purinergic receptor deletion was assessed by multiplex IHC and flow cytometry analyses.

**Figure 1:**
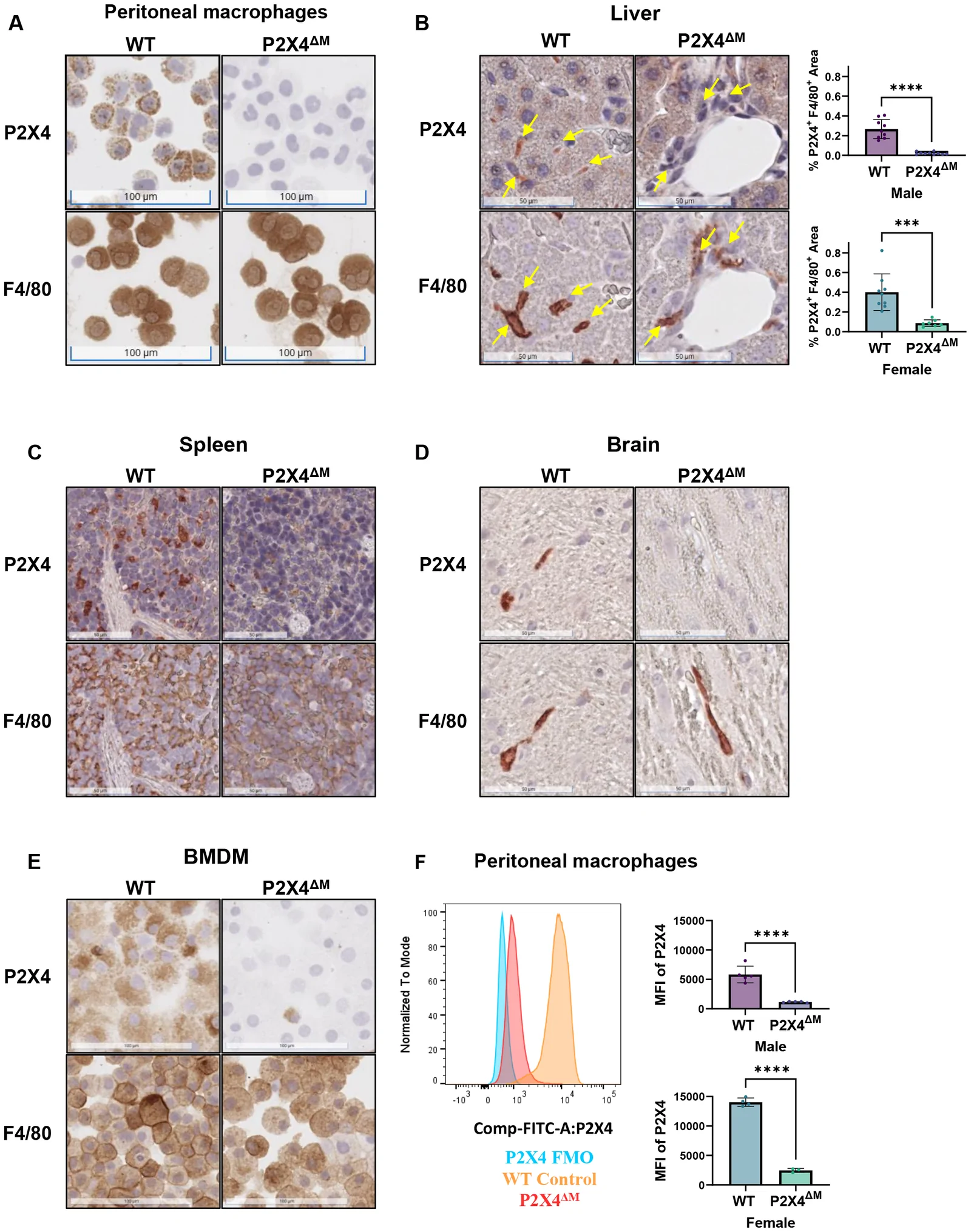
Representative IHC images showing loss of P2X4 purinergic receptor protein expression in **(A)** F4/80^+^ peritoneal cavity macrophages **(B)** F4/80^+^ Kupffer cells, Quantification of the percentage of P2X4 positive area in liver tissue n = 3 biological replicates per group. **(C)** F4/80^+^ splenic red pulp macrophages, **(D)** F4/80^+^ macrophages in brain tissues and **(E)** F4/80^+^ bone marrow–derived macrophages (BMDMs) from P2X4^ΔM^ mice. Yellow arrows indicate F4/80^+^ macrophages. Scale bars, 100 µm **(A, E)** and 50 µm **(B–D)**. **(F)** Flow cytometry analysis demonstrating reduced P2X4 purinergic receptor protein expression in peritoneal cavity macrophages from P2X4^ΔM^ mice compared with WT controls (FMO, Florescence minus one, MFI, Median Florescence Intensity). Data are presented as mean ± SD. n = 4–7 biological replicates per group. Statistical significance was determined using an unpaired t-test (***P < 0.001 ****P < 0.0001).

mIHC analysis demonstrated strong P2X4 purinergic receptor signal in F4/80^+^ macrophages in the peritoneal cavity (**Figure 1A**), liver (**Figure 1B**; **Supplementary Figure 7**), spleen (**Figure 1C**; **Supplementary Figure 8**), brain (**Figure 1D**; **Supplementary Figure 9**) and BMDMs (**Figure 1E**) from WT mice. In contrast, P2X4 purinergic receptor staining was absent in essentially all F4/80^+^ macrophages from the peritoneal cavity (**Figure 1A**), liver (**Figure 1B**), and spleen (**Figure 1C**) and most F4/80^+^ macrophages in brain (**Figure 1D**) and BMDMs (**Figure 1E**) from P2X4^ΔM^ mice. Flow cytometry analysis corroborated a marked loss of P2X4 purinergic receptor signal in peritoneal macrophages from P2X4^ΔM^ mice compared with WT mice (**Figure 1F**).

Together, these findings demonstrate sufficient macrophage-specific loss of the P2X4 purinergic receptor in P2X4^ΔM^ mouse tissues validating the mouse as a useful model to investigate the role of P2X4 purinergic receptors in macrophages across different physiological contexts.

### 3.2 Myeloid-specific P2X4 purinergic receptor loss did not affect overall animal growth but reduced fecundity

Comparable growth rates were observed from 6 to 12 weeks of age between P2X4^ΔM^ and WT mice in both sexes (**Supplementary Figure 3B**). We quantified pup production over a three-month breeding period and found that P2X4^ΔM^ breeding pairs produced significantly fewer male (0.53-fold, p = 0.011) and female (0.51-fold, p = 0.006) offspring per cage compared with WT pairs (**Supplementary Figure 3C**). Lower offspring numbers resulted from both fewer litters (WT-28; P2X4^ΔM^-23) and smaller litter sizes (WT-8.5; P2X4^ΔM^-6.2) from P2X4^ΔM^ breeding pairs compared to controls (p = 0.004) (**Supplementary Figure 3D**).

### 3.3 P2X4 purinergic receptor loss in myeloid cells reduced F4/80^+^ resident macrophage density in liver, spleen and peritoneal tissues

**Figure 2:**
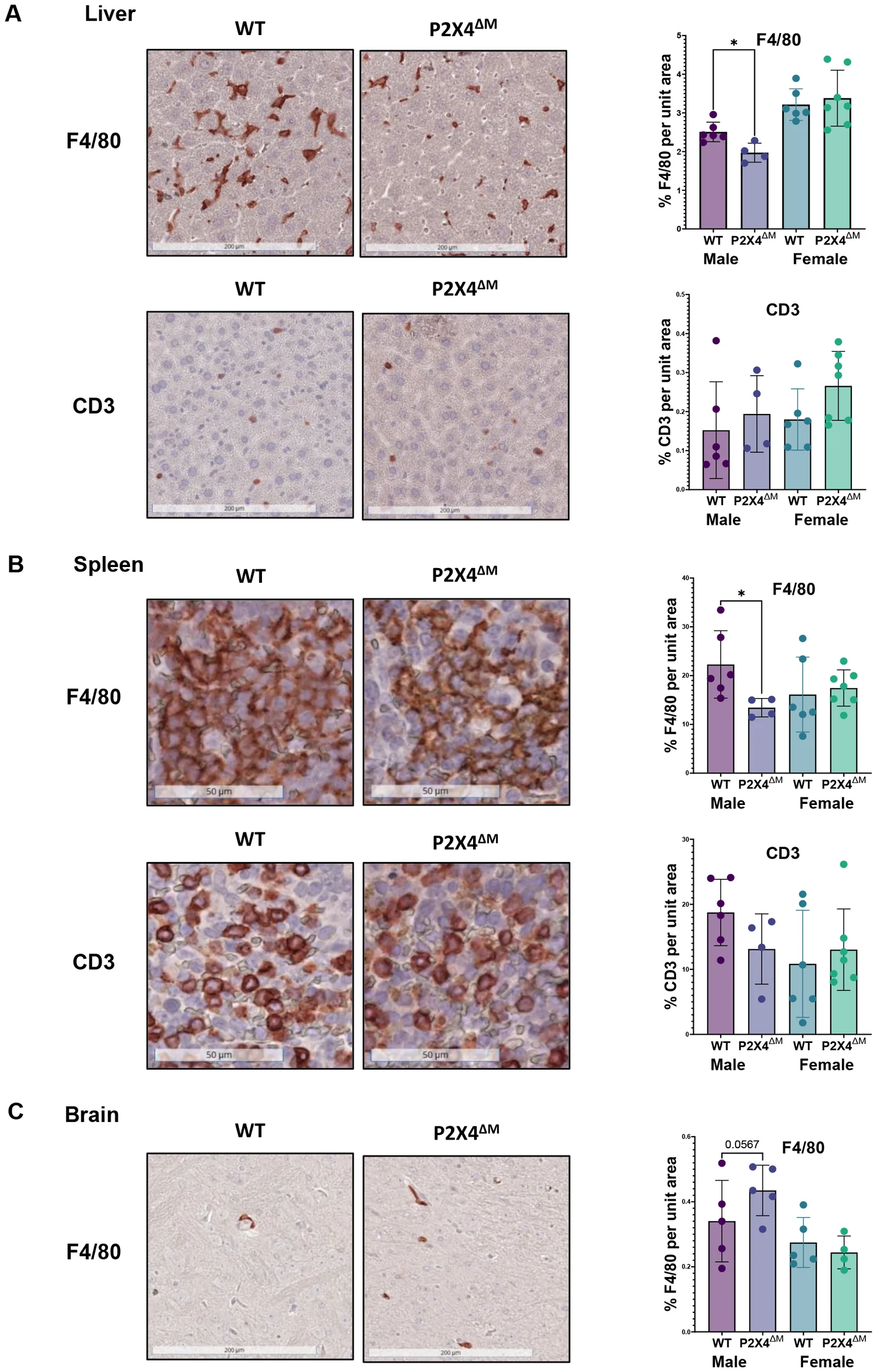
Representative multiplex IHC images and quantitative image analysis of F4/80^+^ macrophages and CD3^+^ T cells in **(A)** liver, **(B)** spleen and **(C)** brain tissues. Scale bars, 200 µm **(A, C)** and 50 µm **(B)**. Data are presented as mean ± SD. n = 4-7 biological replicates per group. Statistical significance was determined using ordinary one-way ANOVA as appropriate (*P < 0.05).

We used multiplex IHC analysis to determine the effect of P2X4 purinergic receptor loss in macrophages on immune cell composition within liver and spleen tissues (**Supplementary Figure 1A**). Quantitative image analysis showed a significant reduction of F4/80^+^macrophages in livers (0.79-fold, p = 0.019), and spleens (0.6-fold, p = 0.0095) from male P2X4^ΔM^ mice compared with male WT mice (**Figures 2A, B**). There were no differences observed in female mice (**Figures 2A, B**). CD3^+^ T cells were comparable between genotypes (**Figures 2A, B**).

Likewise, we used multiplex IHC to assess macrophage distribution in brain tissues from P2X4^ΔM^ mice (**Supplementary Figure 1B**). We observed no P2X4 purinergic receptor protein signal on Iba1^+^P2Y12^+^ microglia (**Supplementary Figure 5**). By contrast, we detected robust P2X4 purinergic receptor expression on Iba1^+^F4/80^+^ macrophages (**Supplementary Figure 6**). Quantitative image analysis of brain tissue trended toward an increase in F4/80^+^ macrophages in male P2X4^ΔM^ compared with WT mice (1.28-fold, p = 0.0567; **Figure 2C**).

To assess the peritoneal immune composition in P2X4^ΔM^ mice, we performed flow cytometric analysis. Live singlets were first identified (**Supplementary Figure 10A**) and sequentially gated to define T cells (CD11b^-^CD3^+^; **Supplementary Figure 10B**), B cells (CD19^+^; **Supplementary Figure 10C**), myeloid cells (CD11b^+^; **Supplementary Figure 11A**), macrophages (CD11b^+^F4/80^high^; **Supplementary Figure 11A**), large peritoneal macrophages (LPMs; CD11b^+^F4/80^high^TIM4^+^; **Supplementary Figure 11A**), small peritoneal macrophages (SPMs; CD11b^+^F4/80^high^TIM4^+^; **Supplementary Figure 11A**), monocytes (CD11b^+^Ly6C^+^F4/80^low/int^Ly6G^+^; **Supplementary Figure 11B**), and neutrophils (CD11b^+^Ly6G^+^F4/80^low/int^; **Supplementary Figure 11C**).

Flow cytometric analysis confirmed that the P2X4 purinergic receptor was highly expressed in macrophages in both sexes (**Figure 1B**, **Supplementary Figure 12**). In contrast, B cells, neutrophils, monocytes and T cells exhibited low to no detectable P2X4 purinergic receptor protein (**Supplementary Figure 12**). LysMcre targets myeloid cells, but its recombination efficiency varies across different myeloid populations and is reported to be highest in macrophages (24, 25). Consistent with this, we observed a robust reduction of P2X4 purinergic receptor expression in peritoneal macrophages, whereas peritoneal monocytes and neutrophils did not show a notable decrease, likely reflecting lower Cre-mediated recombination efficiency and their relatively low baseline expression of P2X4 purinergic receptor (**Supplementary Figure 12**). As expected, no reduction in P2X4 purinergic receptor expression was observed in B cells or T cells, which do not express LysMcre (**Supplementary Figure 12**).

**Figure 3:**
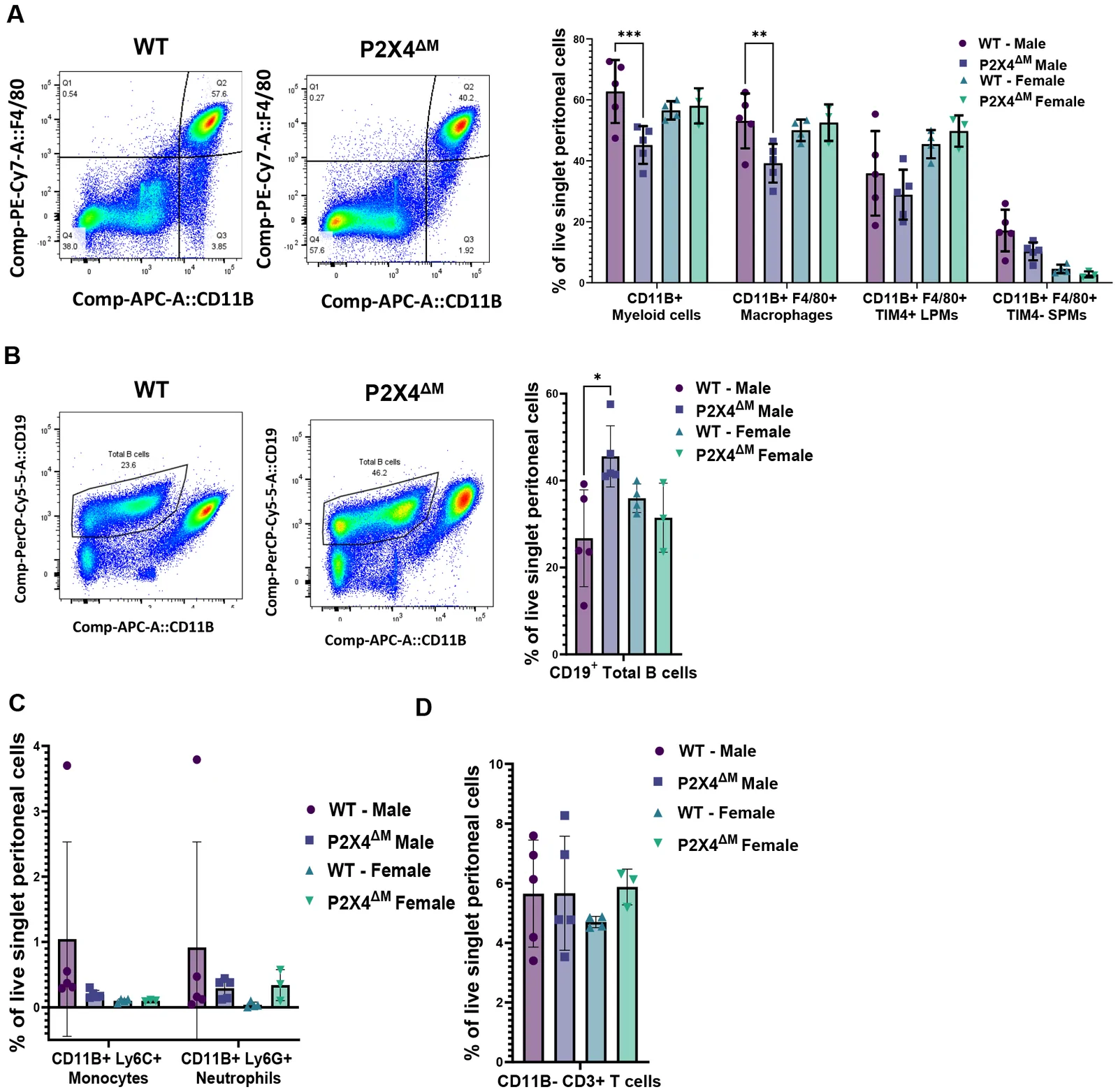
Representative flow cytometry plots and quantification of **(A)** CD11b^+^ myeloid cells, CD11b^+^F4/80^high^ macrophages, large peritoneal macrophages (LPMs; CD11b^+^F4/80^high^TIM4^+^), and small peritoneal macrophages (SPMs; CD11b^+^F4/80^high^TIM4^-^), **(B)** CD19^+^ B cells, **(C)** CD11b^+^Ly6C^+^ monocytes and CD11b^+^Ly6G^+^ neutrophils, and **(D)** CD11b^-^CD3^+^ T cells. Data are presented as mean ± SD. n = 3–7 biological replicates per group. Statistical significance was determined by unpaired t test, or two-way ANOVA as appropriate (*P < 0.05, **P < 0.01, ***P < 0.001).

Notably, there were significantly reduced percentages of CD11b^+^ myeloid cells (0.72-fold, p = 0.0003) and CD11b^+^F4/80^high^ macrophages (0.74-fold, p = 0.0034) in the peritoneal cavity of P2X4^ΔM^ compared with WT male mice (**Figure 3A**). CD11b^+^F4/80^high^TIM4^+^ LPMs, CD11b^+^F4/80^high^TIM4^-^ SPMs macrophage subsets similarly trended downward in male P2X4^ΔM^ mice but were not statistically significant (**Figure 3A**). There was no macrophage differences observed in females (**Figure 3A**). CD19^+^ total B cells were significantly increased in P2X4^ΔM^ compared to WT male mice (1.7-fold, p = 0.0028), whereas no difference was observed among female mice (**Figure 3B**). The percentages of CD11b^+^Ly6C^+^ monocytes, CD11b^+^Ly6G^+^ neutrophils (**Figure 3C**), and CD11b^-^CD3^+^ T cells were comparable between P2X4^ΔM^ and WT mice in both sexes (**Figure 3D**).

### 3.4 The P2X4 purinergic receptor was the predominant P2 purinergic receptor expressed in murine macrophages

**Figure 4:**
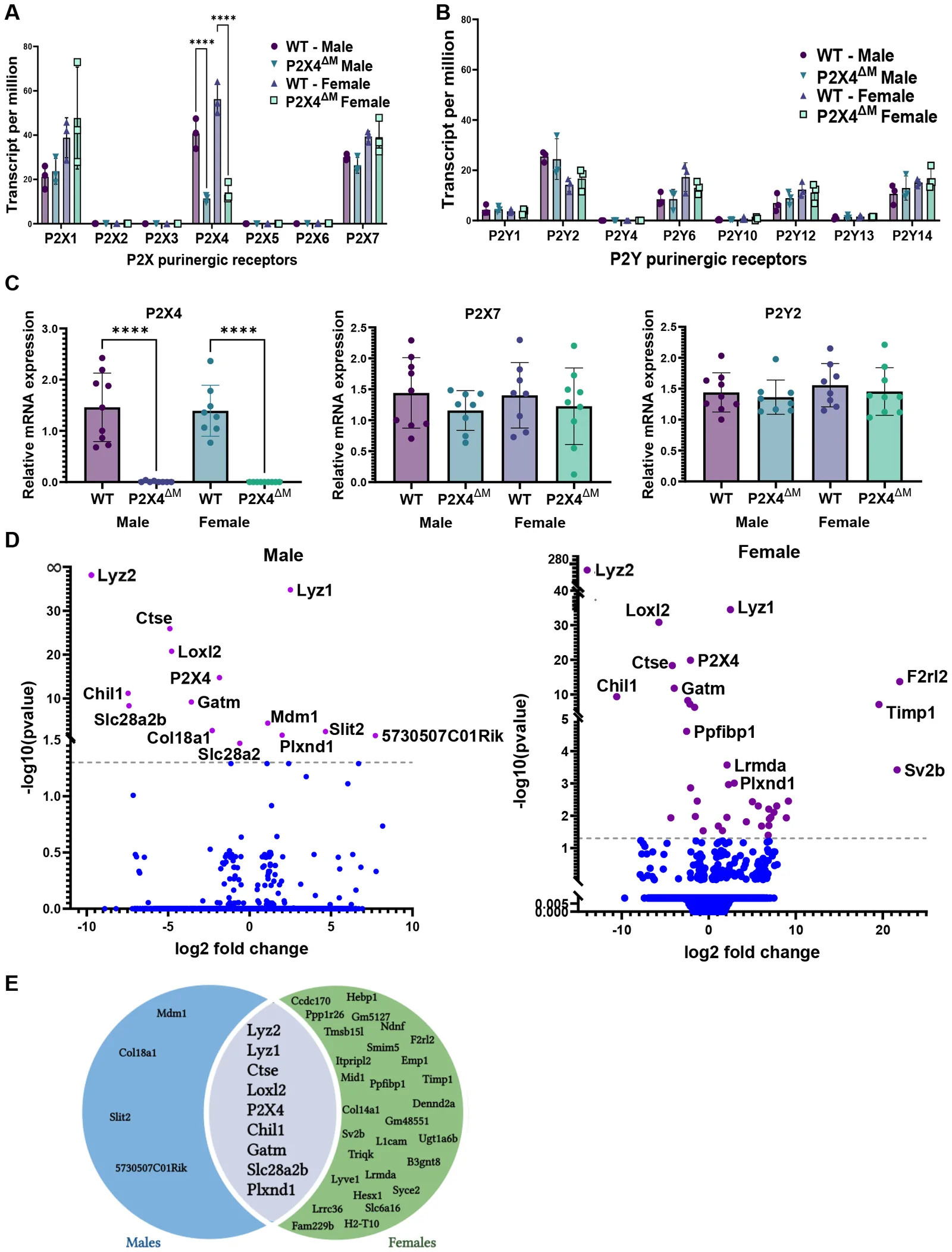
RNA-seq analysis of **(A)** P2X and **(B)** P2Y purinergic receptor mRNA expression in peritoneal macrophages isolated from male and female WT and P2X4^ΔM^ mice. Data are presented as normalized transcript counts. n = 3 biological replicates per group. **(C)** qRT-PCR analysis of P2X4, P2X7, and P2Y2 purinergic receptor expression in BMDMs from male and female WT and P2X4^ΔM^ mice. Gene expression was normalized to GAPDH and calculated using the ΔΔCt method. Data are presented as mean ± SD. n = 8–9 biological replicates per group. Statistical significance was determined using two-way ANOVA or ordinary one-way ANOVA as appropriate (****P < 0.0001). **(D)** Volcano plots showing differentially expressed genes (DEGs) in peritoneal macrophages from male and female P2X4^ΔM^ mice compared with WT controls. Each dot represents an individual gene plotted by log2 fold change (x-axis) versus −log10(P value) (y-axis). Significantly upregulated genes are shown in purple (adjusted P < 0.05). **(E)** Venn diagram illustrating overlapping and sex-specific differentially expressed genes between male and female P2X4^ΔM^ macrophages compared with their respective WT controls.

We isolated peritoneal cells via lavage and flow sorted to isolate macrophages (CD11b^+^F4/80^high^). RNA was isolated and RNA-seq analysis performed. The P2X4 purinergic receptor was the most abundant among all 15 P2 purinergic receptors (**Figures 4A, B**) in macrophages from WT controls. We measured moderate P2X1 and P2X7, but no P2X2, P2X3, P2X5, and P2X6 purinergic receptor expression in WT controls (**Figure 4A**). Among P2Y purinergic receptors, P2Y2, P2Y6, P2Y12, and P2Y14 purinergic receptors were moderately expressed. P2Y1 and P2Y13 purinergic receptors had low expression while P2Y4, and P2Y10, purinergic receptors were below our detectable threshold of 1 TPM (**Figure 4B**).

RNA-seq analysis corroborated a significant reduction in P2X4 purinergic receptor expression in macrophages from P2X4^ΔM^ compared with WT from both male and female mice (p = <0.0001, **Figure 4A**). We validated P2X4 purinergic receptor loss using qRT-PCR on RNA isolated from BMDMs (p = <0.0001, **Figure 4C**). There was no obvious transcript per million (TPM) alterations in any other P2 purinergic receptors in macrophages from P2X4^ΔM^ mice, supporting the specificity of the knockout and ruling out likely compensatory P2 purinergic receptor changes. To validate this observation, we used qRT-PCR to measure P2X7 and P2Y2 mRNA expression, which are often co-expressed in macrophages and implicated in overlapping signaling pathways (26–28). Both receptors were comparably expressed between genotypes in both sexes (**Figure 4C**), further validating the model as useful for the specific study of P2X4 purinergic receptors without the likely influence of other P2 purinergic receptors at baseline.

### 3.5 P2X4 purinergic receptor deletion induced more transcriptional changes in macrophages from female compared to male mice

We performed differential gene expression analysis of RNA-seq data from peritoneal macrophages to determine the transcriptional consequence of P2X4 purinergic receptor deletion. Lysozyme 2 (*Lyz2*), lysozyme 1 (*LyZ1*), Cathepsin E (*Ctse*), *Loxl2*, P2X4, Chitinase-3-like 1 (*Chil1*), Glycine amidinotransferase (Gatm), Solute Carrier Family 28 Member 2b (*Slc28a2b*), and *Plxnd1* were differentially expressed between genotypes in macrophages from both male and female mice (**Figures 4D, E**). In addition to the common genes described above, there were three additional differentially expressed genes (DEGs) in males and twenty-nine additional DEGs in females (**Figures 4D, E**). This disparity suggests that P2X4 purinergic receptor signaling may have a more extensive regulatory role in macrophages from female mice.

Lyz2 was significantly reduced in both males and females (**Figure 4D**). This decrease likely reflects the use of the Lyz2-Cre system and does not represent a downstream biological response. Correspondingly, Lyz1 was strongly upregulated and among the most prominently changed genes in both sexes. Given its similar function to Lyz2, this increase is likely a compensatory response to maintain lysosomal or antimicrobial activity in the absence of LyZ2.

### 3.6 P2X4 purinergic receptor deletion resulted in enrichment of interferon-β response and angiogenesis-related pathways in macrophages from male mice

**Figure 5:**
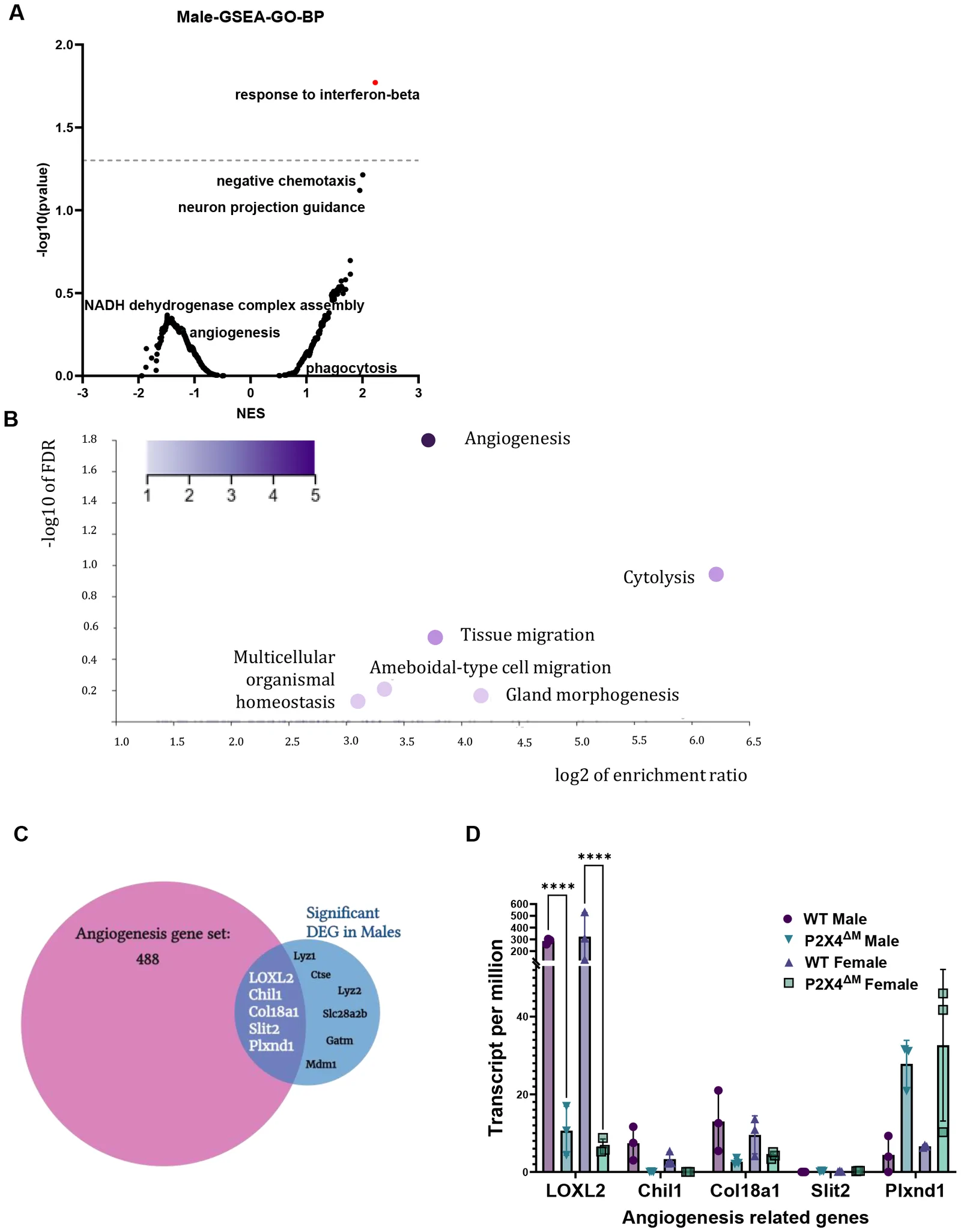
GSEA and ORA of DEGs from P2X4^ΔM^ macrophages in males. **(A)** GSEA results plotted as normalized enrichment score (NES) on the x-axis and −log10(adjusted P value) on the y-axis for significantly enriched Gene Ontology (GO) Biological Process (GO-BP), horizontal dashed line indicates the significance threshold (adjusted P = 0.05). Selected representative enriched terms are labeled, and red points indicate significantly enriched terms. **(B)** GO ORA of the top enriched biological processes among significant DEGs, plotted as log2(enrichment ratio) on the x-axis and −log10(FDR) on the y-axis. **(C)** Venn diagram showing overlap between the angiogenesis gene set (GO:0001525; n = 488) and significantly DEGs (n = 11) identified in peritoneal macrophages from male mice. **(D)** RNA-seq expression analysis of angiogenesis-related genes in peritoneal macrophages from WT and P2X4^ΔM^ mice stratified by sex. Data are presented as transcripts per million (TPM). Data are presented as mean ± SD (****P < 0.0001). n = 3 biological replicates per group.

We performed gene set enrichment analysis (GSEA) on ranked gene lists to identify biological processes associated with the transcriptional changes in macrophages from P2X4^ΔM^ mice. Pathways related to the response to IFN- β were significantly enriched in macrophages from male P2X4^ΔM^ mice (p = 0.017, **Figure 5A**). Gene Ontology over-representation analysis (ORA) of the DEGs in macrophages from male mice identified significant enrichment of the angiogenesis gene set (GO:0001525; enrichment ratio = 12.96; p < 0.0001; FDR = 0.016; **Figure 5B**).

### 3.7 P2X4 purinergic receptor deletion reduced *Loxl2* and increased *Plxnd1* expression in macrophages

Among the overlapping DEGs between male and female mice, we observed a set of tissue remodeling and angiogenesis related genes (**Figure 4E**). Among males, there were 5 genes that overlap with the Gene Ontology angiogenesis gene set (GO:0001525), specifically, *Loxl2*, *Chil1*, collagen type XVIII alpha 1 chain (*Col18a1*), Slit guidance ligand 2 (*Slit2*), and *Plxnd1* (**Figure 5C**). Similarly, *Loxl2*, *Chil1* and *Plxnd1* were DEGs among females that overlap with the angiogenesis gene set.

*Loxl2* expression, which promotes angiogenesis (29), was significantly reduced in macrophages from P2X4^ΔM^ compared to WT from male (p adj = 1.76E-15) and female (p adj = 1.54E-31; **Figure 5D**) mice. Pro-angiogenic *Chil1* expression was also reduced in male mice (p adj = 6.53E-12) but this trend was not statistically significant among female mice (p adj = 4.48E-10). In contrast, *Plxnd1* expression, which negatively regulates angiogenesis, was increased in macrophages from both male (p adj = 0.0238) and female (p adj = 0.0011) P2X4^ΔM^ mice. *Col18a1* was reduced in male P2X4^ΔM^ mice (p adj = 0.0021) and trended downward in macrophages from female P2X4^ΔM^ mice (p adj = 0.7374). *Slit2* expression was below our detectable threshold of 1 TPM (**Figure 5D**).

**Figure 6:**
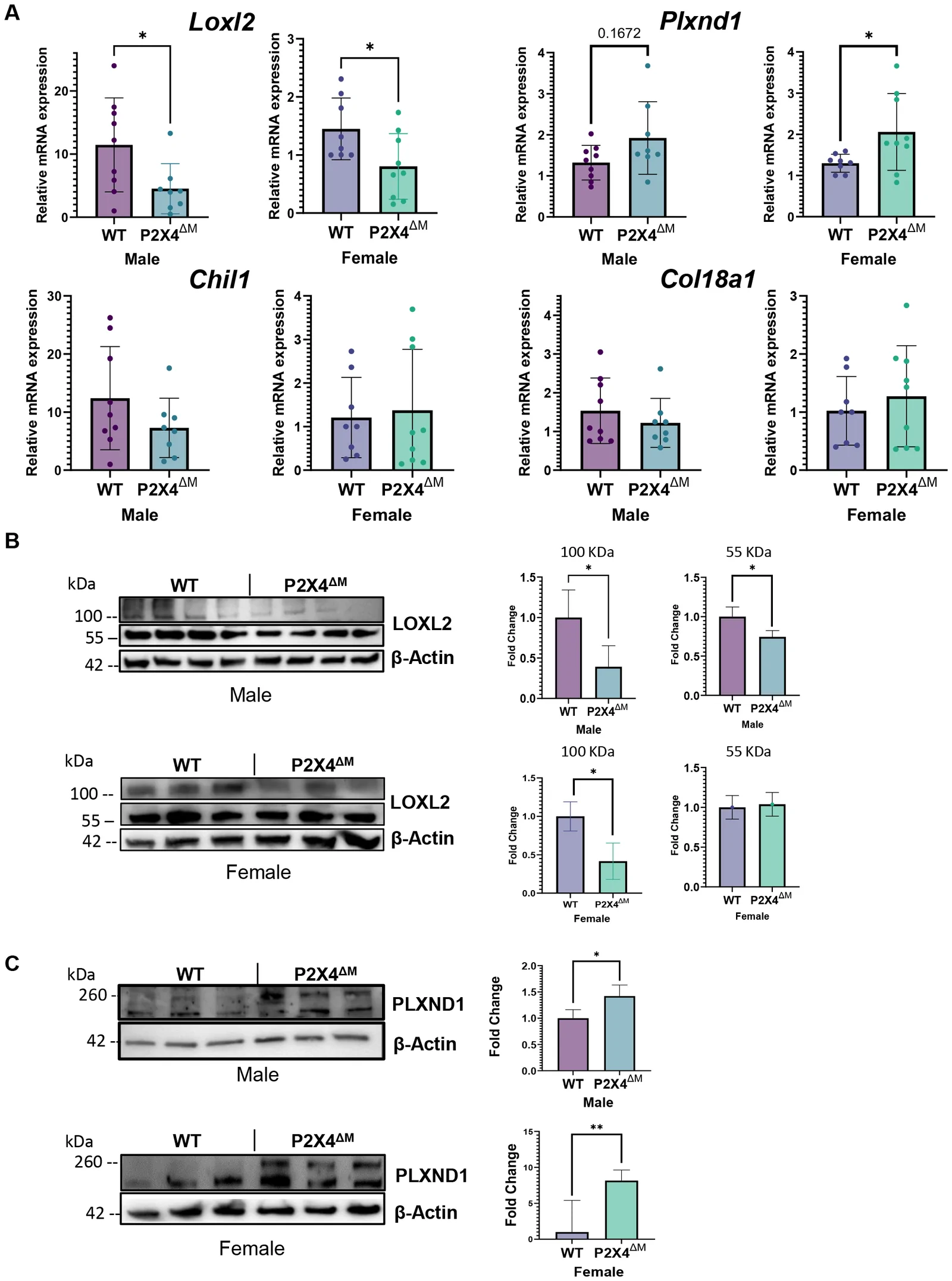
Validation of angiogenesis-related transcriptional and protein changes in macrophages from P2X4^ΔM^ mice. **(A)** qRT-PCR validation of *Loxl2*, *Plxnd1*, *Chil1*, and *Col18a1* mRNA expression in bone marrow–derived macrophages from WT and P2X4^ΔM^ mice stratified by sex. Gene expression was normalized to GAPDH and calculated using the ΔΔCt method. Data are presented as mean ± SD. n = 8–9 biological replicates per group. Western blot analysis showing expression and corresponding densitometric quantification of **(B)** LOXL2 protein and **(C)** PLXND1 protein in BMDMs from WT and P2X4^ΔM^ mice. β-Actin was used as a loading control. Data are presented as mean ± SD. n = 4 biological replicates per group. Statistical significance was determined using the Mann–Whitney U test or unpaired t test as appropriate (*P < 0.05, **P < 0.01).

We validated these RNA-seq data by qRT-PCR using RNA isolated from BMDMs (**Figure 6A**). Reduced *Loxl2* mRNA expression was confirmed in BMDMs from both male (p = 0.0464) and female (p = 0.0334) P2X4^ΔM^ mice (**Figure 6A**). *Plxnd1* mRNA expression was significantly increased in macrophages from female P2X4^ΔM^ mice (p = 0.0345), with a similar but non-significant trend in male mice (**Figure 6A**). Chil1 mRNA expression trended similarly to the RNA-seq data in male mice and there was no apparent change in *Col18a1* mRNA expression between genotypes (**Figure 6A**).

We further analyzed LOXL2 and PLXND1 changes using western blotting. Consistent with the qRT-PCR, LOXL2 protein was significantly reduced in macrophages from female P2X4^ΔM^ mice (0.4-fold, p = 0.03; **Figure 6B**). We also noted that processed LOXL2, detected at 55kDa, which is thought to be the catalytically active fragment of LOXL2 (30, 31) was significantly reduced in macrophages from male P2X4^ΔM^ mice (0.74-fold, p = 0.0132; **Figure 6B**). PLXND1 protein was increased in macrophages from male (2.37-fold, p = 0.03) and female (8.15-fold, p = 0.002) P2X4^ΔM^ mice (**Figure 6C**). Together, these results suggest that deletion of the P2X4 purinergic receptor in macrophages may alter how macrophages influence angiogenesis processes.

### 3.8 P2X4 purinergic receptor deletion was associated with enrichment of metabolic pathways related gene sets in female macrophages

**Figure 7:**
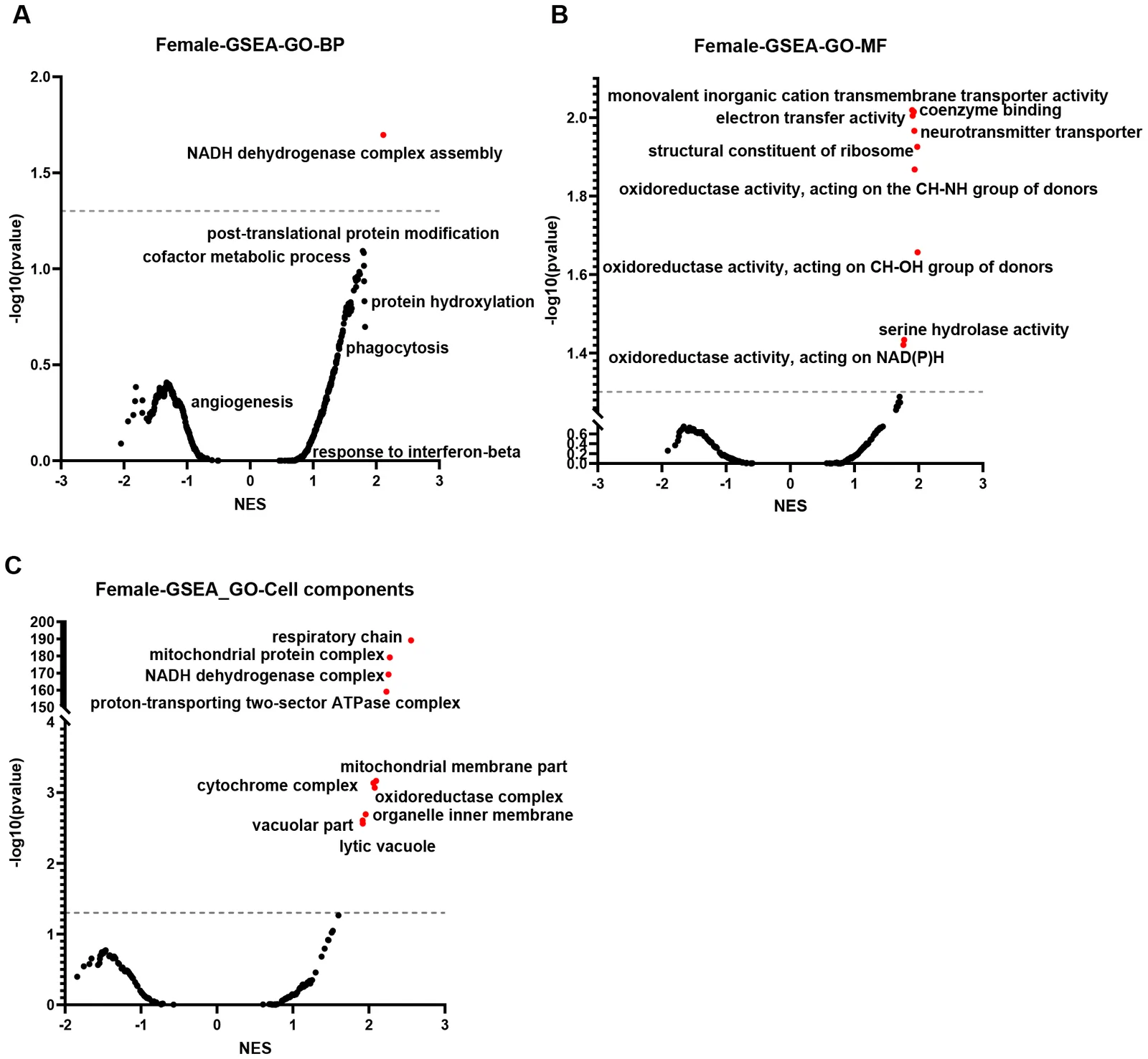
GSEA and ORA of DEGs from P2X4^ΔM^ macrophages in females. Panels **(A-C)** show GSEA results plotted as normalized enrichment score (NES) on the x-axis and −log10(adjusted P value) on the y-axis for significantly enriched Gene Ontology (GO) categories. **(A)** GO-BP enrichment in female **(B)** GO Molecular Function (GO-MF) enrichment in female mice. **(C)** GO Cellular Component (GO-CC) enrichment in female mice. In each panel, the horizontal dashed line indicates the significance threshold (adjusted P = 0.05). Selected representative enriched terms are labeled, and red points indicate significantly enriched terms.

GSEA biological process analysis showed enrichment of NADH dehydrogenase complex assembly genes (p = 0.02) (**Figure 7A**) in macrophages from female P2X4^ΔM^ mice. Like macrophages from male mice, angiogenesis-related pathways were negatively enriched in female P2X4^ΔM^ mice, but this was not statistically significant (**Figure 7A**). GSEA molecular function analysis showed enrichment of pathways including oxidoreductase activity, ion transfer activity, and coenzyme binding (**Figure 7B**). GSEA cellular component analysis showed enrichment of mitochondrial protein complexes and respiratory chain components (**Figure 7C**). Together these results demonstrate enrichment of mitochondrial and metabolic pathways in macrophages from female P2X4^ΔM^ mice. Gene Ontology ORA of the DEGs in macrophages from female mice did not identify any significantly enriched pathways.

### 3.9 P2X4 purinergic receptor deletion reduced CIV-MTCO1 and HSP60 expression in female macrophages

**Figure 8:**
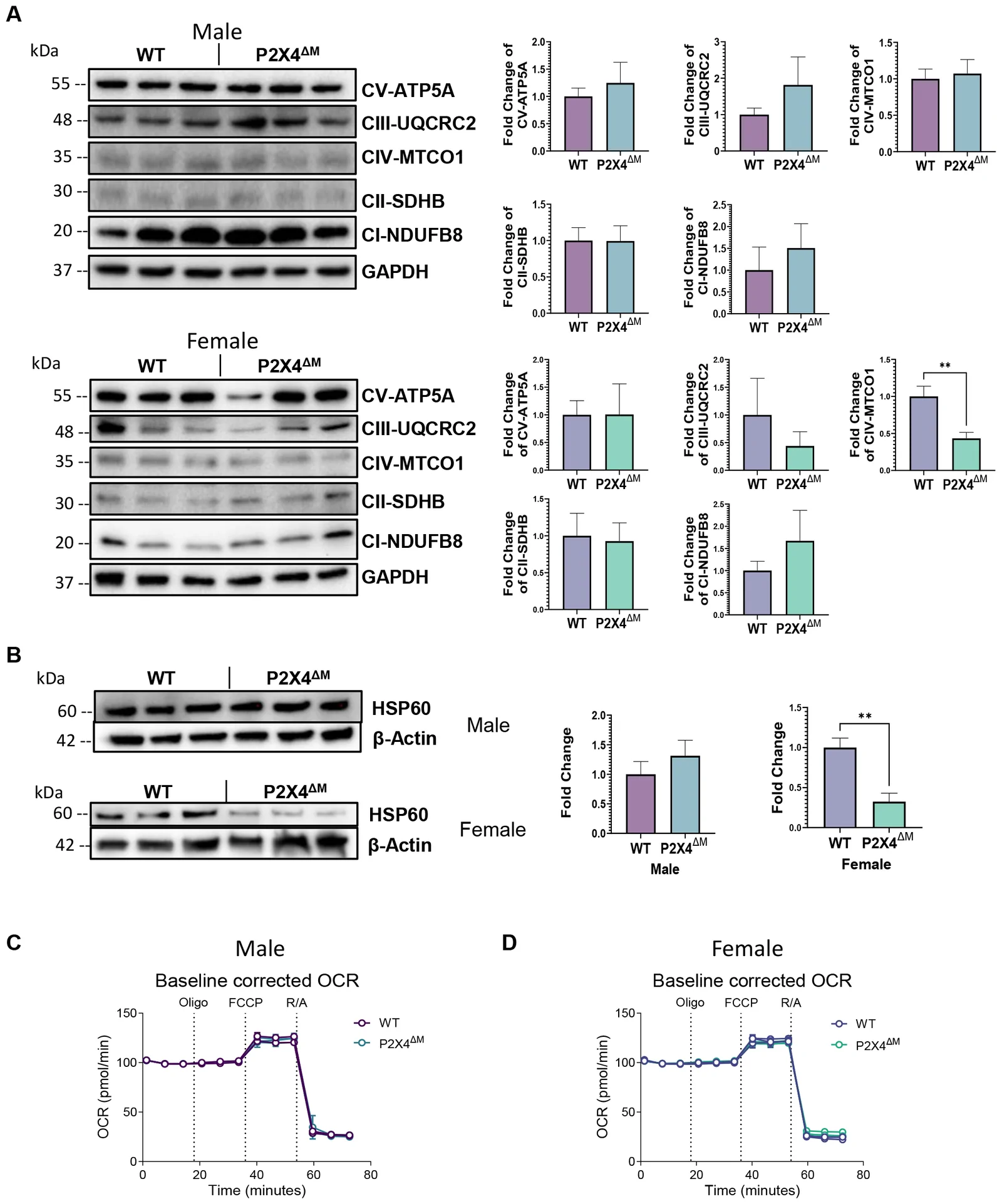
Western blot analysis showing expression and corresponding densitometric quantification of **(A)** OXPHOS complex and **(B)** HSP60. β-Actin was used as a loading control. Data are presented as mean ± SD. n = 3 biological replicates per group. Statistical significance was determined using the unpaired t test (**P < 0.01). Representative baseline-corrected oxygen consumption rate (OCR) traces of BMDMs from WT and P2X4^ΔM^ **(C)** male and **(D)** female mice. OCR was monitored following sequential injections of oligomycin (Oligo), FCCP, and rotenone/antimycin A (R/A). n = 3 biological replicates per group.

To determine whether the enrichment of mitochondrial and oxidative phosphorylation pathways was associated with changes in mitochondrial respiratory chain components, we quantified the mitochondrial OXPHOS complex, and HSP60. There were no significant differences in the expression of OXPHOS complex proteins or HSP60 between WT and P2X4^ΔM^ male macrophages (**Figures 8A, B**). In contrast, macrophages from female P2X4^ΔM^ mice showed a significant reduction in CIV-MTCO1 protein expression compared with WT controls (0.43-fold, p = 0.0036; **Figure 8A**). Expression levels of CI-NDUFB8, CII-SDHB, CIII-UQCRC2, and CV-ATP5A were not altered. Further, HSP60 protein expression was significantly reduced in macrophages from female P2X4^ΔM^ mice (0.32-fold, p = 0.0018; **Figure 8B**).

### 3.10 Oxygen consumption rate profiles are largely unchanged in P2X4^ΔM^ and WT

To determine whether the enrichment of mitochondrial and oxidative phosphorylation pathways was associated with altered mitochondrial respiration, OCR was measured using a Seahorse extracellular flux assay. Representative baseline-corrected OCR traces were comparable between WT and P2X4^ΔM^ BMDMs following sequential injection of oligomycin, FCCP, and rotenone/antimycin A. OCR increased in response to FCCP and decreased following rotenone/antimycin A treatment in macrophages from both male and female mice, indicating intact mitochondrial respiratory responses (**Figures 8C, D**).

### 3.11 P2X4 purinergic receptor deletion attenuated LPS-induced IL-6 and MCP-1 secretion in macrophages

**Figure 9:**
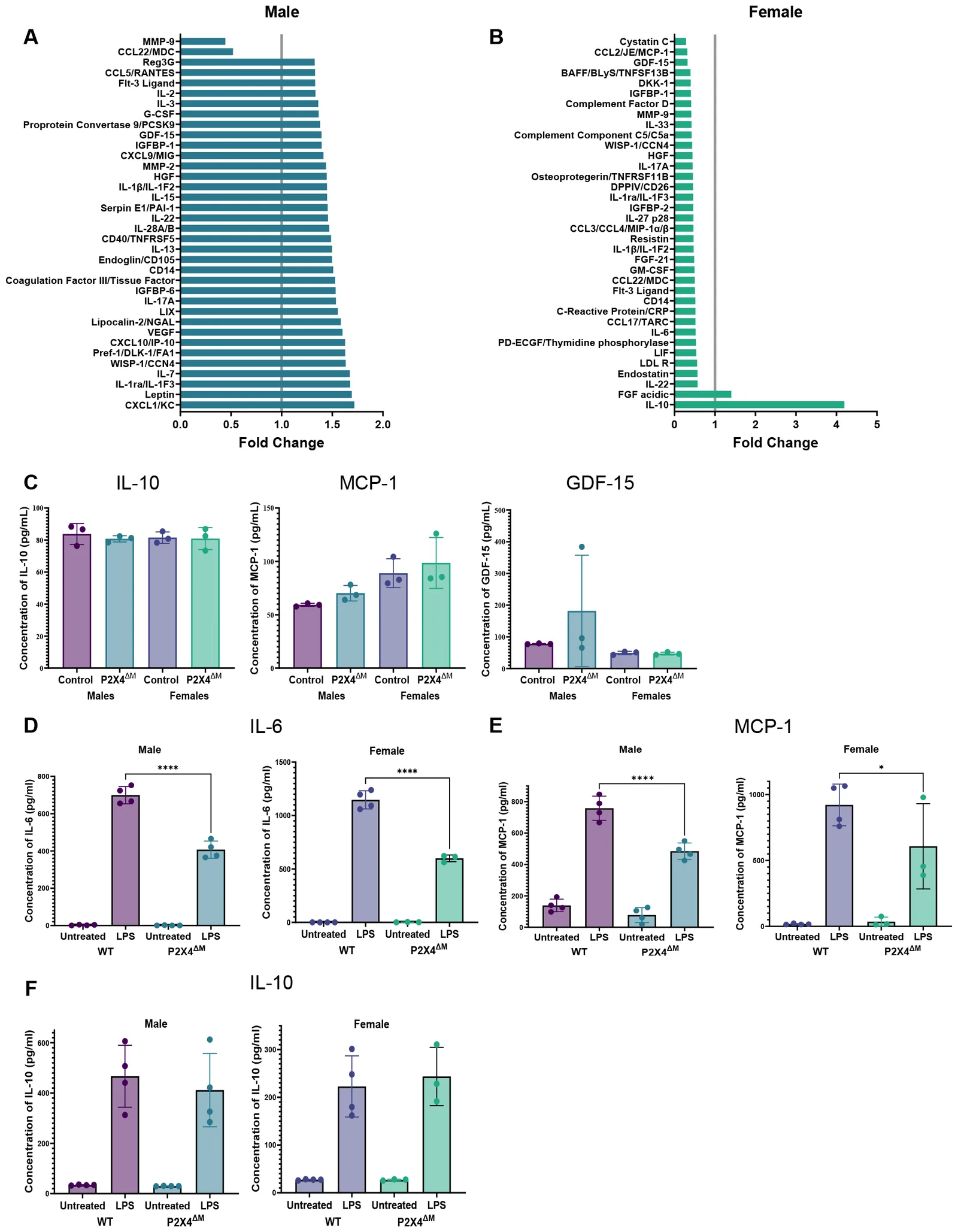
**(A)** Fold change in serum cytokines and growth factors levels in **(A)** male and **(B)** female P2X4^ΔM^ mice relative to WT. Cytokine levels of **(C)** IL-10, MCP-1, and GDF-15 in serum collected from WT and P2X4^ΔM^ mice. n = 3 biological replicates per group. Cytokine secretion from BMDMs isolated from WT and P2X4^ΔM^ mice under untreated (un) and overnight LPS (20 ng/mL) stimulation conditions: **(D)** IL-6, **(E)** MCP-1, and **(F)** IL-10. n = 3–4 biological replicates per group. Data are presented as mean ± SD. Statistical significance was determined using ordinary one-way ANOVA (*P < 0.05, ****P < 0.0001).

To assess the functional impact of P2X4 purinergic receptor deletion on macrophage secretion, we assessed the systemic cytokine levels in serum from WT and P2X4^ΔM^ mice using a membrane-based antibody array which analyses 111 cytokines, chemokines, and growth factors (**Supplementary Figure 13**, **14**). Among male mice, Matrix Metalloproteinase-9 (MMP-9, 0.44-fold) was reduced while Platelet-Derived Endothelial Cell Growth Factor (PD-ECGF, 2.68-fold), interleukin-6 (IL-6, 2.45-fold), Leukemia Inhibitory Factor (LIF, 2.40-fold), Cystatin C (2.09-fold), and 20 other factors were increased ≥1.5-fold in serum from P2X4^ΔM^ compared to WT mice (**Figure 9A**). Among female mice, Cystatin C (0.29-fold), Monocyte Chemoattractant Protein-1 (MCP-1, 0.32-fold), Growth Differentiation Factor 15 (GDF-15, 0.32-fold), and 20 other factors were reduced <0.5-fold while only IL-10 (4.19-fold) was increased in serum from P2X4^ΔM^ compared to WT mice (**Figure 9B**). ELISA analysis determined that IL-10, MCP-1, and GDF-15 concentrations were comparable between genotypes in both male and female mice (**Figure 9C**), indicating no significant differences under basal conditions. 18h LPS stimulation induced robust IL-6, MCP1, and IL-10 secretion in BMDMs from both WT and P2X4^ΔM^ mice. However, IL-6 secretion was significantly attenuated in BMDMs from P2X4^ΔM^ mice compared with WT mice in both sexes (Male: 0.58-fold, p = 0.0001; Female: 0.52-fold, p = 0.00001; **Figure 9D**). Similarly, LPS-mediated MCP-1 secretion was significantly attenuated in BMDMs from male and female P2X4^ΔM^ mice compared with WT mice (Male: 0.64-fold, p = 0.0011; Female: 0.66-fold, p = 0.0356; **Figure 9E**). LPS stimulation induced comparable IL-10 secretion between macrophages from WT and P2X4^ΔM^ mice (**Figure 9F**).

### 3.12 P2X4 purinergic receptor deletion enhances macrophage phagocytic activity

**Figure 10:**
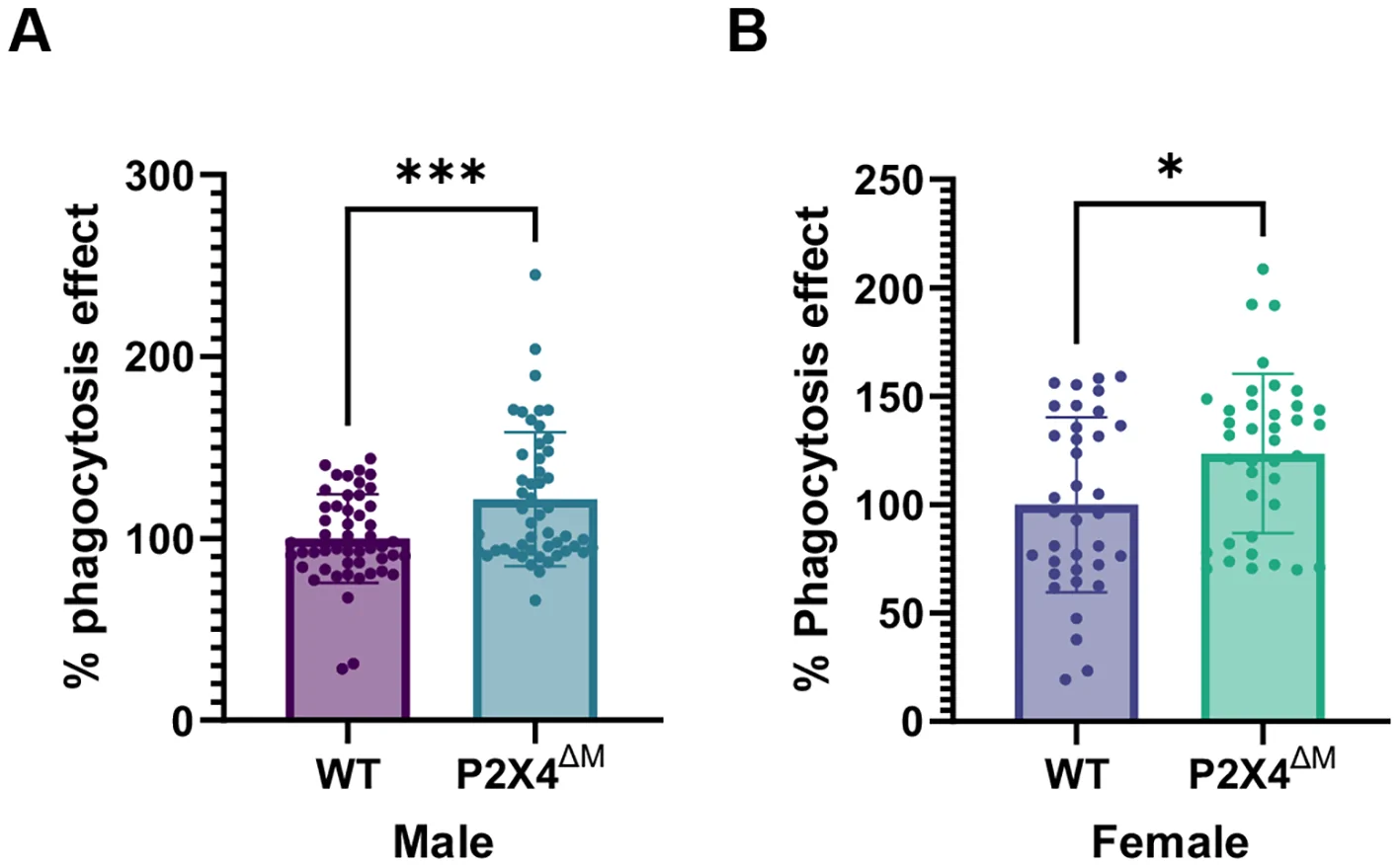
Phagocytic activity was assessed using fluorescently labeled Escherichia coli bioparticles in BMDMs from WT and P2X4^ΔM^ mice **(A)** male and **(B)** female mice. Phagocytic activity is expressed as percentage of the WT control. Data are presented as mean ± SD. n = 4 biological replicates per group. Statistical significance was determined using an unpaired t-test (*P < 0.05, ***P < 0.001).

To determine whether P2X4 deficiency influences macrophage phagocytic function, phagocytosis efficiency was assessed in BMDMs from WT and P2X4^ΔM^ mice. P2X4^ΔM^ macrophages exhibited significantly enhanced phagocytic activity compared with WT controls in both sexes (**Figures 10A, B**). In males, phagocytosis increased by approximately 21.5% in P2X4^ΔM^ mice relative to WT cells (P = 0.0009; **Figure 10A**). Similarly, female P2X4^ΔM^ mice showed an approximately 23.6% increase in phagocytic activity compared with WT macrophages (P = 0.0103; **Figure 10B**). These findings indicate that P2X4 purinergic receptors play a role in controlling macrophage phagocytic activity.

## 4 Discussion

In this study, we generated and characterized a P2X4^ΔM^ mouse. Using a combination of multiplex IHC, flow cytometry, and transcriptomic analyses, we confirmed efficient deletion of P2X4 purinergic receptors in macrophages across different tissues and examined the consequence on immune cell distribution, biological pathways, and macrophage function.

We confirmed P2X4 purinergic receptor deletion in F4/80^+^ macrophages in the peritoneum, liver, spleen, brain, and BMDM of P2X4^ΔM^ mouse. Few macrophages derived from P2X4^ΔM^ mouse bone marrow maintained P2X4 purinergic receptor expression. BMDM residual P2X4 purinergic receptor expression may be due to the presence of “escaper” cells in *ex vivo* cultures—immature hematopoietic progenitors that fail to undergo Cre-mediated recombination due to low Lyz2 promoter activity (32).

The LysMCre system targets myeloid cell populations but we observed the most dramatic P2X4 purinergic receptor loss in macrophages. Monocytes and neutrophils have considerably less P2X4 purinergic receptor expression compared to macrophages, and there was minimal reduction in P2X4^ΔM^ mice. As expected, no change was observed in B cells or T cells. As such, we present a macrophage-specific P2X4 purinergic receptor knockout mouse suitable for studies specifically investigating the role of P2X4 purinergic receptors in macrophage populations *in vivo*.

We report comparable growth rates in P2X4^ΔM^ mice compared to WT mice. However, there was reduced fecundity in P2X4^ΔM^ mice. A study demonstrated that inflammatory macrophage populations play important roles in ovarian aging and impaired reproductive function (33). Further investigation is required to determine whether P2X4 purinergic receptor loss alters immune regulation or tissue homeostasis that influences reproductive outcomes.

A consistent finding across tissues was a reduction in macrophage density in male P2X4^ΔM^ mice. This was observed in liver, spleen, and the peritoneal cavity suggesting that P2X4 purinergic receptor signaling may contribute to tissue-resident macrophage homeostasis under basal conditions. Future studies will be aimed at determining whether this reduced density is due to reduced macrophage recruitment, proliferation, differentiation, or survival. It is reported that extracellular ATP maintained the viability of murine IL-3-dependent myeloid progenitor 32D cells by preventing programmed cell death through autocrine survival loops (34). Therefore, loss of the high-sensitivity ATP–gated P2X4 purinergic receptor may reduce responsiveness to essential ATP-mediated survival signals (35). Impaired responsiveness may hinder the replenishment and maintenance of mature macrophages, leading to reduced populations as we observed in P2X4^ΔM^ tissues. Subsequently, reduced macrophage populations may contribute to the increased B cell percentages quantified in P2X4^ΔM^ mice. Peritoneal macrophages suppress B cell proliferation and activation through cytokine production and suppressive mediators such as prostaglandin, which helps keep B cells in a quiescent state (36). Future studies will be aimed at investigating the role of P2X4 purinergic receptors in macrophage-mediated B cell homeostasis.

Contrary to our findings in other tissues, the density of F4/80^+^ macrophages trended upward in brain tissues. P2 purinergic receptors are critical regulators of microglial function. Previous studies have demonstrated that, in the healthy brain, metabotropic P2Y12 purinergic receptors are highly expressed and serve as key markers of microglial homeostasis (37, 38). Upon microglial activation, P2Y12 purinergic receptor expression decreases, while purinergic receptors such as P2X4 and P2X7 become upregulated and contribute to the secretion of pro-inflammatory mediators (38). In the present study, Iba1^+^P2Y12^+^ microglial cells did not express P2X4 purinergic receptors. Our findings are consistent with previous reports indicating that P2X4 purinergic receptor expression remains relatively low under homeostatic conditions and is primarily induced during pathological or inflammatory activation states (37, 39, 40). Accordingly, the P2X4 purinergic receptor signal detected in Iba1^+^F4/80^+^ populations indicates that these cells are likely infiltrating macrophages, as they were primarily observed in or near blood vessels. A few Iba1^+^F4/80^+^ may also be activated microglia.

We observed sex differences in the transcriptional response to P2X4 purinergic receptor deletion. Macrophages from female mice had a larger number of DEGs compared with macrophages from male mice, suggesting that P2X4 purinergic receptor signaling may be more broadly integrated into regulatory networks in female mice. While sex differences in macrophage responses are well described (41–43), this finding indicates that P2X4 purinergic receptor signaling pathways may also be differentially regulated between sexes, which should be considered in future studies. Although this is the first transcriptomic analysis of P2X4 purinergic receptor deletion in macrophages, previous studies have reported sex-dependent functional roles of P2X4 purinergic receptor in various cell types across different pathological contexts (44–47). For instance, P2X4 purinergic receptor signaling is essential for pain hypersensitivity in male rat spinal microglia but not in females (44–46). In stroke models, P2X4 purinergic receptor deletion reduces injury in female but not in male mice (47).

Pathway analysis further supported sex-specific differences. GSEA analysis in males showed that P2X4 purinergic receptor deletion in macrophages was associated with an increased response to IFN-β. IFN-β is a macrophage-derived type I interferon that bridges innate and adaptive immunity by promoting antigen-presenting cell maturation and directing T- and B-cell responses (48). It also supports antiviral defense (49) and helps resolve inflammation by accelerating the resolution phase, including promoting neutrophil apoptosis and clearance of inflammatory cells (50). A recent study indicated that the P2X4 purinergic receptor normally drives IFN-β production through the mtDNA–cGAS–STING axis (51). Perhaps the increased response to IFN-β observed in our study demonstrates a compensatory response to reduced IFN-β production caused by P2X4 purinergic receptor loss.

On the other hand, macrophages from female P2X4^ΔM^ mice had an enrichment of mitochondrial and metabolic pathways, including oxidative phosphorylation and electron transport processes. Metabolic reprogramming is a key determinant of macrophage function, and alterations in mitochondrial pathways may impact macrophage activation states, survival and functional outputs (52). To determine whether these transcriptional changes were associated with alterations in mitochondrial electron transport chain components, we assessed the expression of OXPHOS complexes. Female P2X4^ΔM^ macrophages showed significantly reduced expression of the Complex IV subunit MTCO1. MTCO1 is the final and oxygen accepting complex of the mitochondrial respiratory chain and is responsible for catalyzing electron transfer to molecular oxygen (53). As a key regulator and rate limiting step of oxidative phosphorylation (53), reduced MTCO1 expression may indicate altered mitochondrial respiratory chain homeostasis. To determine whether these molecular alterations were accompanied by functional changes in mitochondrial respiration, we performed Seahorse extracellular flux analysis. Despite the reductions in MTCO1 expression, WT and P2X4^ΔM^ BMDMs exhibited similar OCR profiles at baseline. We also observed reduced HSP60 protein expression in female P2X4^ΔM^ macrophages. HSP60 is a mitochondrial chaperone protein that resides within the mitochondrial matrix and plays a critical role in maintaining mitochondrial proteostasis by facilitating the proper folding, assembly, and maintenance of mitochondrial proteins (54). HSP60 is also commonly upregulated in response to mitochondrial stress (55). Reduced HSP60 expression may reflect altered mitochondrial homeostasis, which is consistent with the enrichment of mitochondrial and oxidative phosphorylation pathways identified by RNA-seq analysis. However, further studies are needed to fully characterize the influence of P2X4 purinergic receptors on macrophage mitochondrial homeostasis, particularly beyond baseline conditions.

Extracellular ATP functions as a danger-associated signal that promotes macrophage activation through P2X4 purinergic receptor signaling and calcium influx (56). Consequently, loss of P2X4 purinergic receptor may reduce ATP-mediated calcium-dependent activation signaling in macrophages. Ca^2+^ influx is essential for mitochondrial respiration and the activity of key metabolic enzymes (57). Tissue-resident macrophage maintenance is highly dependent on mitochondrial fitness and metabolic adaptability, which support long-term survival and self-renewal of tissue-resident macrophages (58). The loss of P2X4 purinergic receptor signaling likely alters mitochondrial function, prompting a compensatory shift in cellular metabolism. This compensatory mechanism was observed only in females, potentially due to the influence of estrogen which enhances mitochondrial function and promotes oxidative metabolism (59, 60).

Metabolic differences may also contribute to the reduced tissue-resident macrophage populations observed in male mice. In macrophages from female mice, enrichment of mitochondrial and metabolic pathways suggests the presence of a compensatory mechanism that supports cellular survival despite P2X4 purinergic receptor loss. In contrast, male macrophages do not exhibit a similar metabolic adaptation, potentially resulting in impaired bioenergetic capacity and reduced survival or retention of macrophages within tissues. Consequently, P2X4 purinergic receptor deficiency may lead to a decline in tissue-resident macrophage populations in males, whereas female mice maintain macrophage populations through metabolically driven compensatory mechanisms. Further studies are required to validate this hypothesis.

Within both sexes, RNA-seq data analysis identified multiple angiogenesis-related DEGs. Reduced expression of *Loxl2*, *Chil1*, and *Col18a1*, together with increased *Plxnd1*, indicates a shift from active vessel growth to a more restricted and stabilized state (61–66). LOXL2 is an extracellular matrix (ECM) enzyme that cross-links collagen and elastin, providing the structural scaffolding necessary for both fibrotic progression and angiogenic sprouting (67). In the context of angiogenesis, LOXL2 promotes endothelial cell migration and the assembly of the vascular basement membrane; its upregulation is frequently associated with increased vessel density (62, 67–69). LOXL2 is also associated with poor prognosis in several types of cancers where it promotes invasion, metastasis, and cancer-associated fibrosis (70, 71). Previous studies demonstrated that CHIL1 promotes the production of pro-angiogenic chemokines and cytokines (63, 72). COL18A1 has context-dependent roles in angiogenesis. As a basement membrane collagen, COL18A1 contributes to vascular structural integrity and extracellular matrix organization, which can support vessel formation (66). However, COL18A1 is also the precursor of endostatin, a well-known anti-angiogenic fragment (64). Therefore, the reduction of *Col18a1* in P2X4^ΔM^ macrophages cannot be interpreted as simply pro- or anti-angiogenic without further analysis. PLXND1 is a transmembrane receptor for class 3 semaphorins and has been identified as a mechanosensor in vascular endothelial cells, with emerging evidence suggesting related regulatory roles in macrophages (73, 74). Semaphorin-PlexinD1 signaling represses angiogenesis by inhibiting VEGF-mediated pro-angiogenic activity (61). Therefore, increased *plxnd1* expression suggests suppression of angiogenesis. These findings are particularly relevant in the context of the TME, where macrophages play key roles in angiogenesis and tumor progression. Overall, our findings indicate that P2X4 purinergic receptors alter the expression of angiogenesis associated pathway genes in macrophages. Future studies will aim to determine whether reduced *Loxl2* and increased *Plxnd1* mRNA and protein expression observed in P2X4^ΔM^ mice may decrease ECM remodeling, limit angiogenesis, and attenuate tumor development.

We investigated the role of P2X4 purinergic receptors in macrophage cytokine secretion. We reported comparable MCP-1, IL-10, or GDF-15 serum levels in P2X4^ΔM^ and WT mice, suggesting that P2X4 purinergic receptor signaling is unlikely to be involved in maintaining systemic cytokine levels under basal conditions.

However, during inflammatory stimulation, we reported significantly attenuated secretion of IL-6, a key mediator of the systemic acute-phase response, and MCP-1, an essential chemokine for monocyte recruitment, in macrophages from P2X4^ΔM^ mice. This is particularly relevant in the TME, as IL-6 and MCP-1 are known drivers of tumor growth, angiogenesis, the recruitment of additional immunosuppressive myeloid cells, and poor prognosis of patients with cancer (75–79). Importantly, the absence of changes in IL-10 levels indicates that P2X4 purinergic receptor does not globally suppress macrophage function. Further studies are needed to comprehensively characterize the role of P2X4 purinergic receptor in cytokine secretion across various physiological contexts.

Phagocytosis is a fundamental macrophage function that contributes to pathogen clearance, tissue homeostasis, and the resolution of inflammation (80). Loss of P2X4 purinergic receptor enhanced phagocytic activity in both male and female macrophages, suggesting that P2X4 signaling normally act to suppress macrophage phagocytic activity. Our findings are consistent with a recent study on Ischemic Stroke showing that genetic deletion or pharmacological inhibition of P2X4 enhances phagocytic uptake by macrophages and microglia (81), suggesting that P2X4 signaling may function as a negative regulator of phagocytosis. Further studies are needed to define the signaling pathways and molecular mechanisms through which P2X4 regulates macrophage phagocytosis.

In summary, this study establishes the P2X4^ΔM^ mouse as a robust model for investigating macrophage-specific P2X4 purinergic signaling. The P2X4 purinergic receptor regulates tissue-resident macrophage density, macrophage transcriptional programming, cytokine secretion, phagocytic activity, and the expression of angiogenesis-associated genes. These findings provide a foundation for future studies to define the role of macrophage-specific P2X4 signaling in various disease contexts, including cancer, where macrophages play a critical role in shaping the TME.

## Data Availability

The bulk RNA sequencing data presented in this study is deposited in the NCBI Gene Expression Omnibus (GEO), under the accession number: GSE346257. Other data supporting the conclusions of this article are included in the article and its **Supplementary Material**.
