# Supplementary material for: P2X4 purinergic receptor loss alters macrophage homeostasis, cytokine secretion, and phagocytosis in a myeloid-specific P2X4 purinergic receptor knockout mouse
Source: Front Immunol. 2026 Sep 10;17:1916582. doi: 10.3389/fimmu.2026.1916582 (PMC13600901; doi:10.3389/fimmu.2026.1916582)
Supplement: Supplementary file 1 [file SupplementaryFile1.pdf]

Suppl. Fig. 1

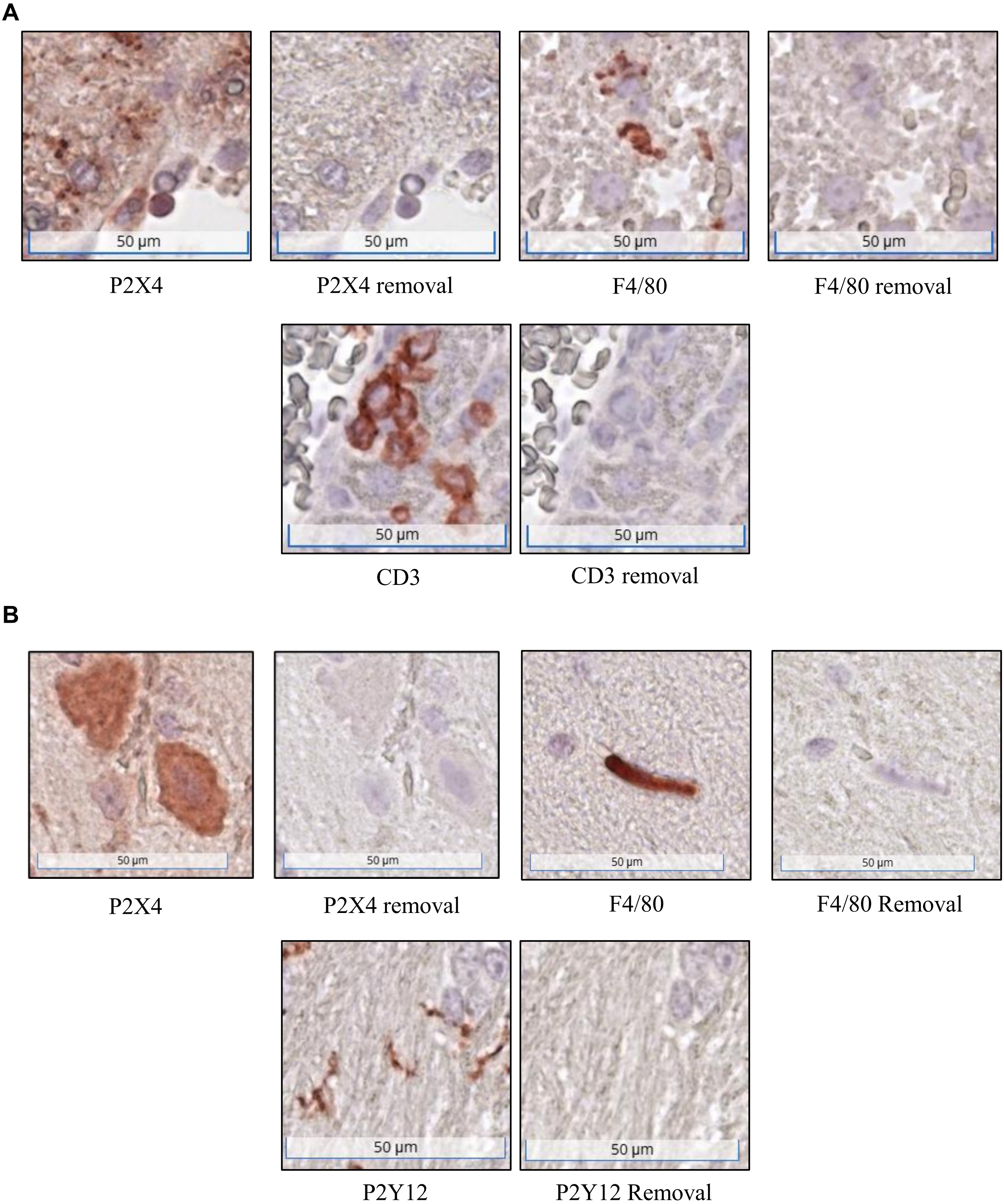
A
50 µm
P2X4
50 µm
P2X4 removal
50 µm
F4/80
50 µm
F4/80 removal
50 µm
CD3
50 µm
CD3 removal
B
50 µm
P2X4
50 µm
P2X4 removal
50 µm
F4/80
50 µm
F4/80 Removal
50 µm
P2Y12
50 µm
P2Y12 Removal

Suppl. Fig. 2

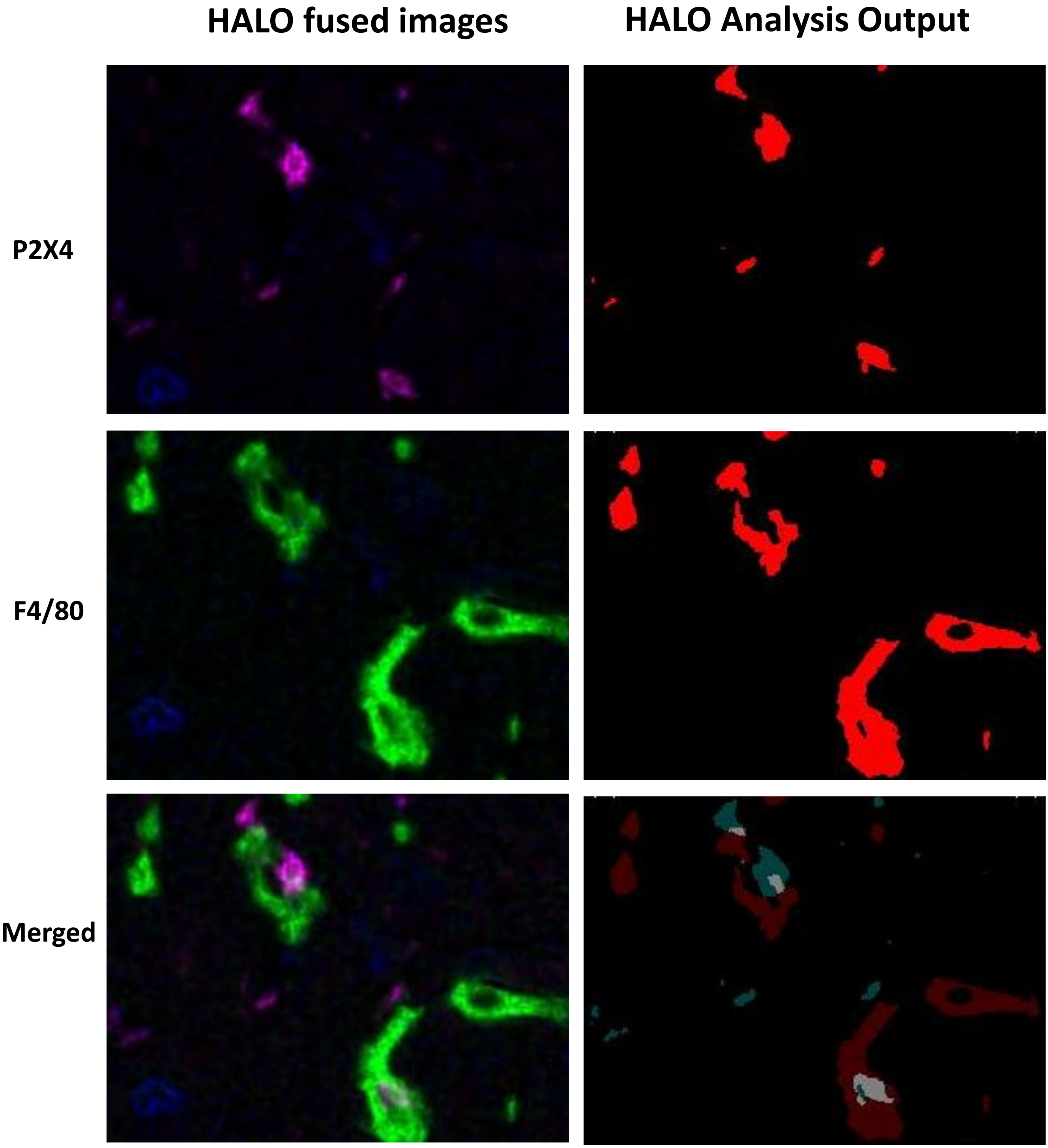

Suppl. Fig. 3

A

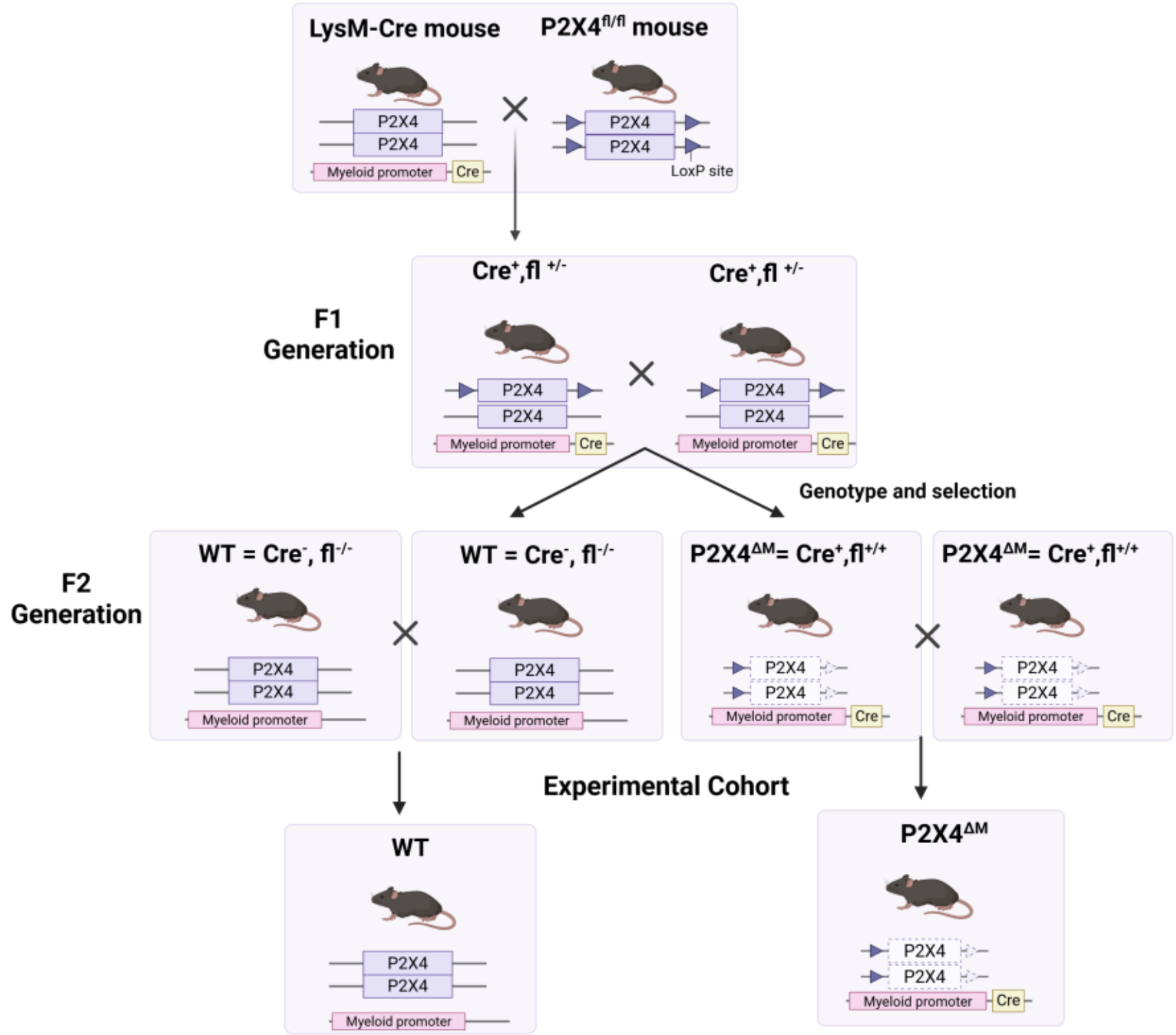

B

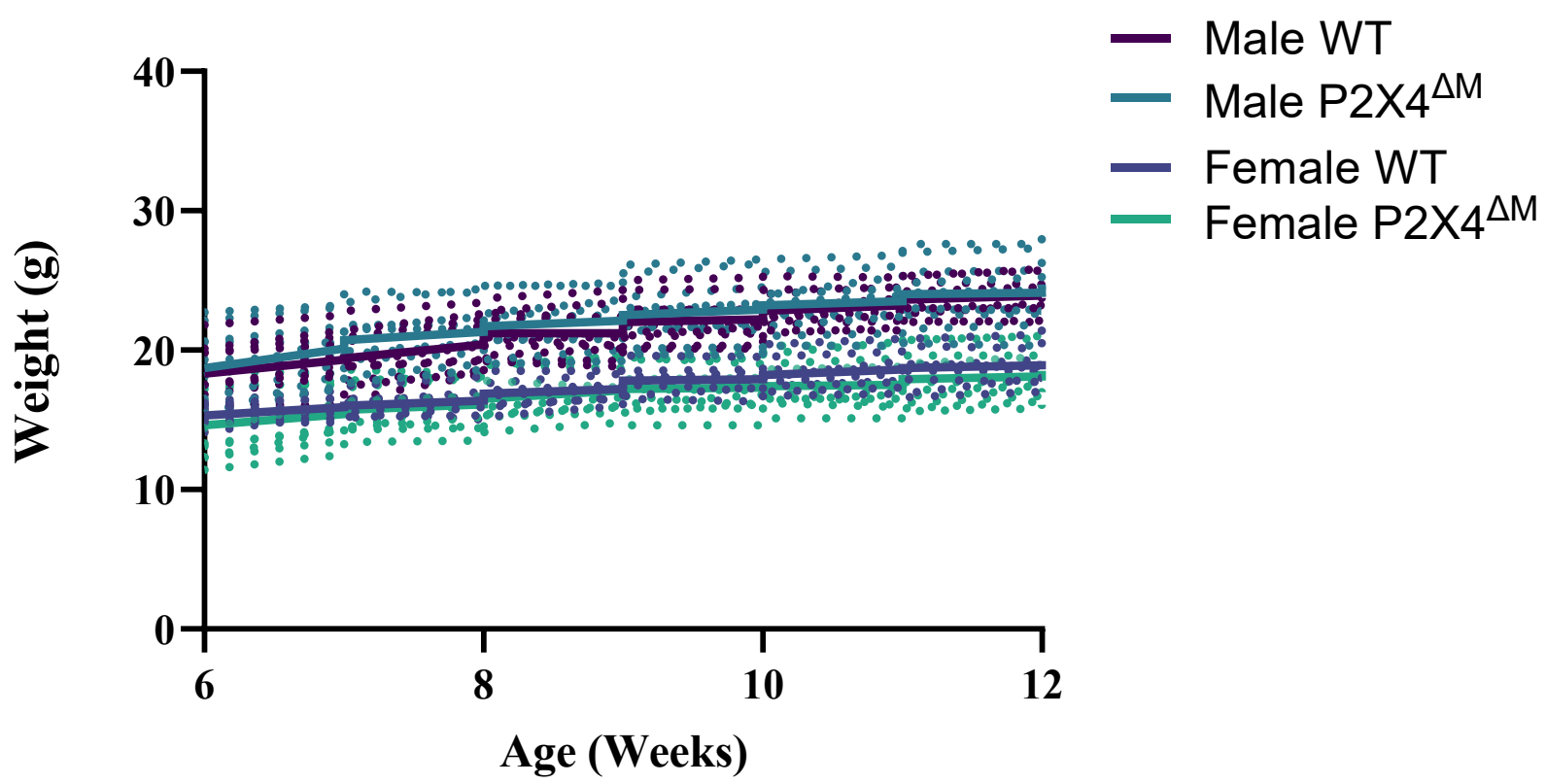

C

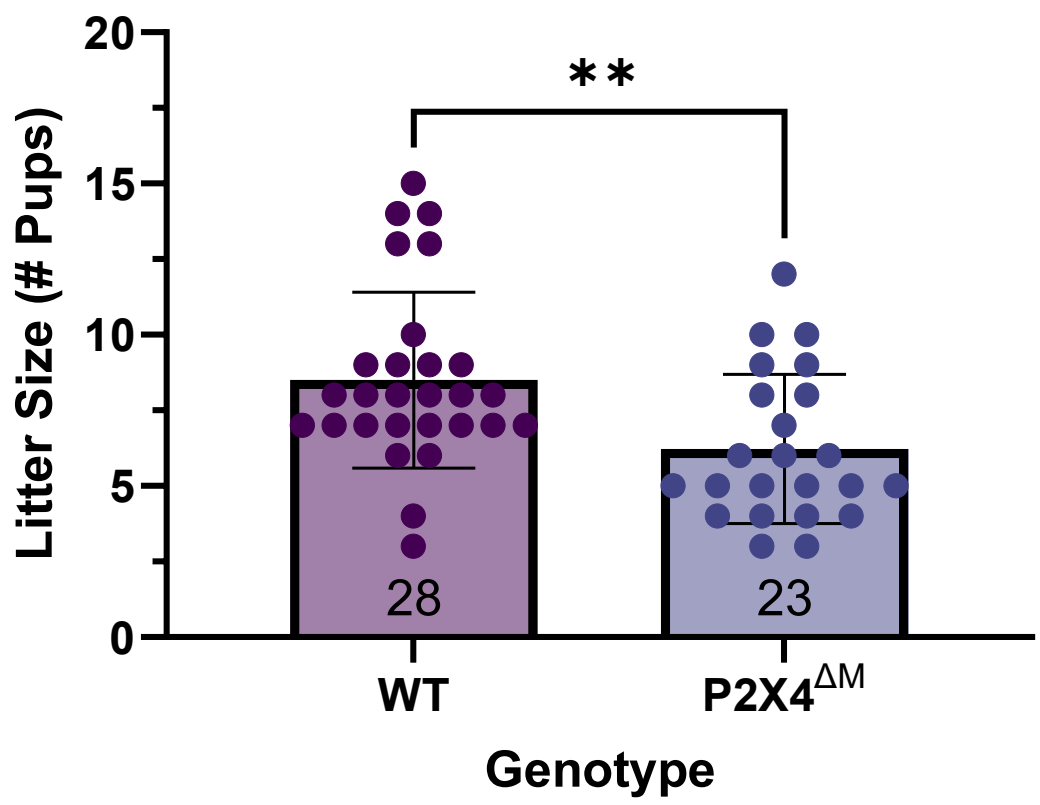

D

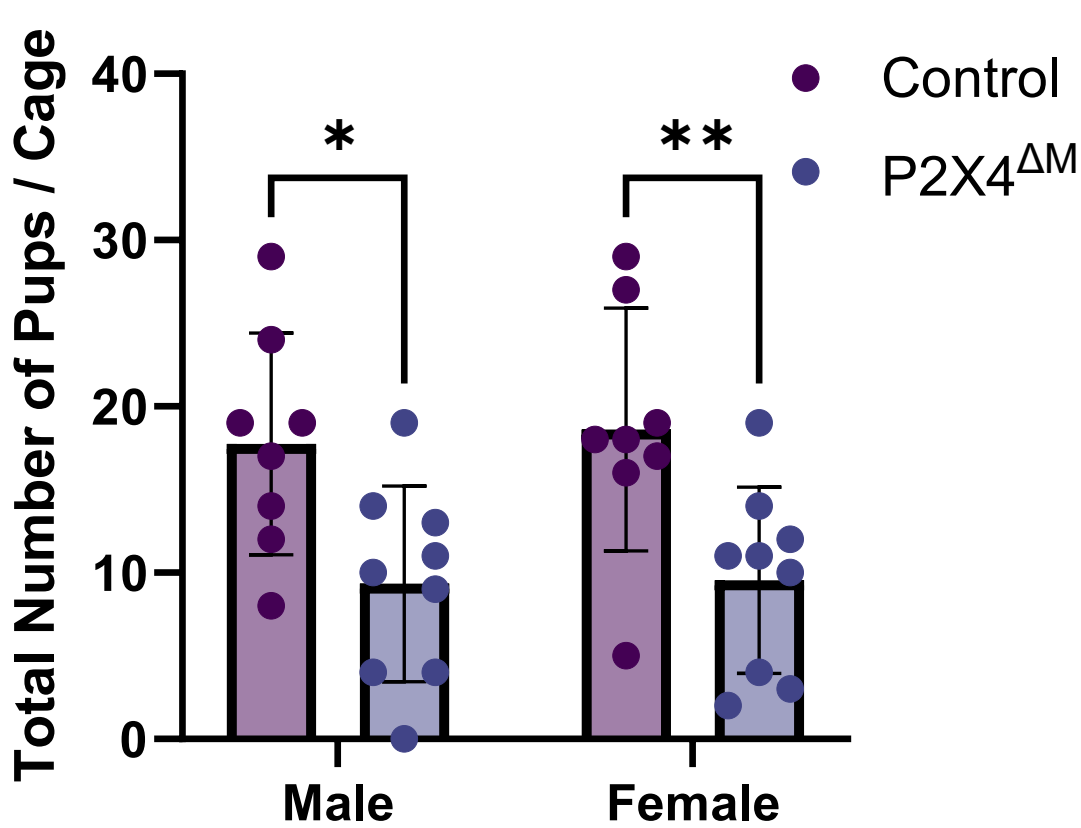

Suppl. Fig. 4

Liver

Floxed (P2X4$^{fl/fl}$; Lyz2-Cre$^{-/-}$) | **WT (P2X4$^{+/+}$; Lyz2-Cre$^{-/-}$)** | **P2X4$^{\Delta M}$ (P2X4$^{fl/fl}$; Lyz2$^{cre/cre}$)**

P2X4

F4/80

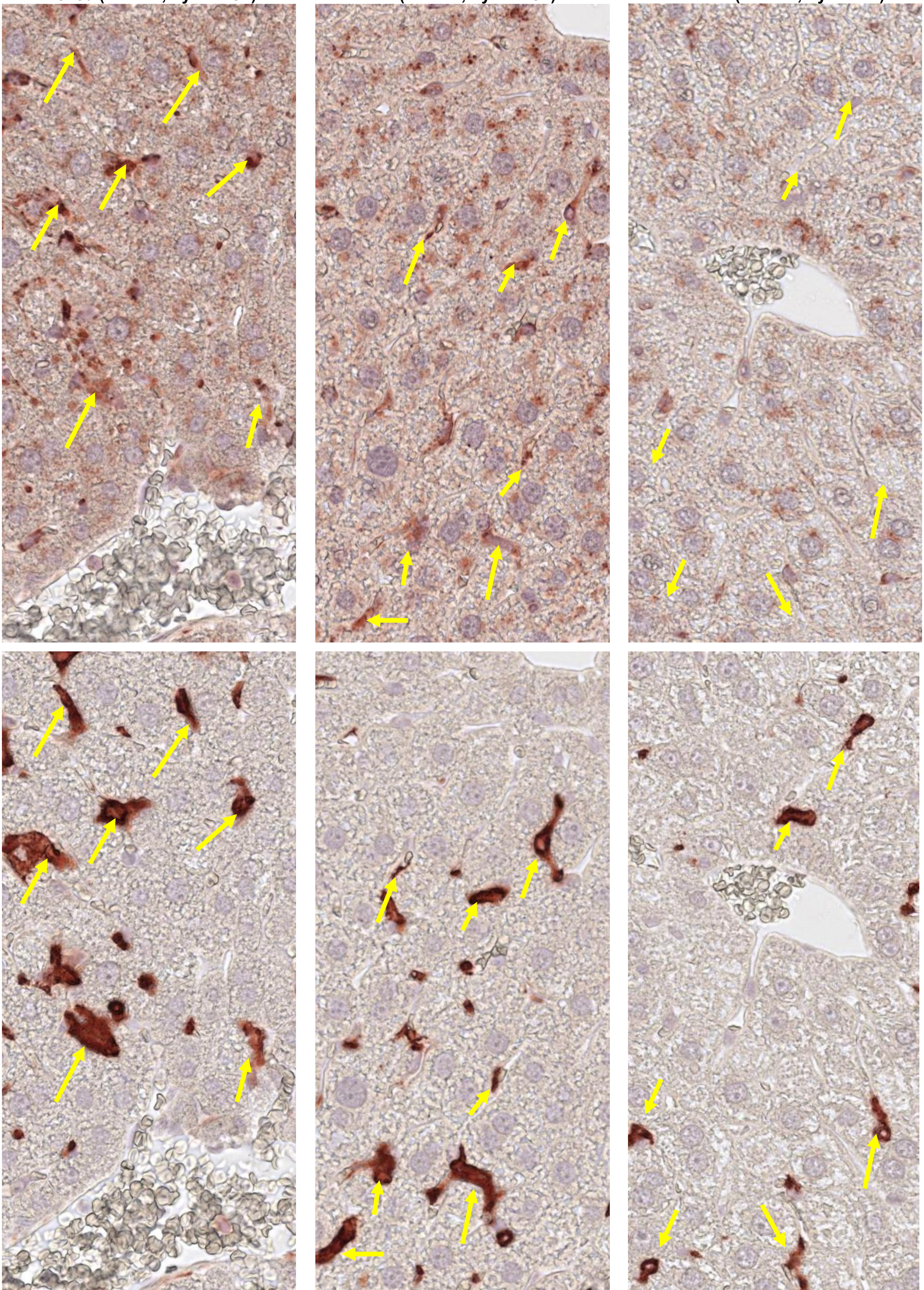

Suppl. Fig. 5

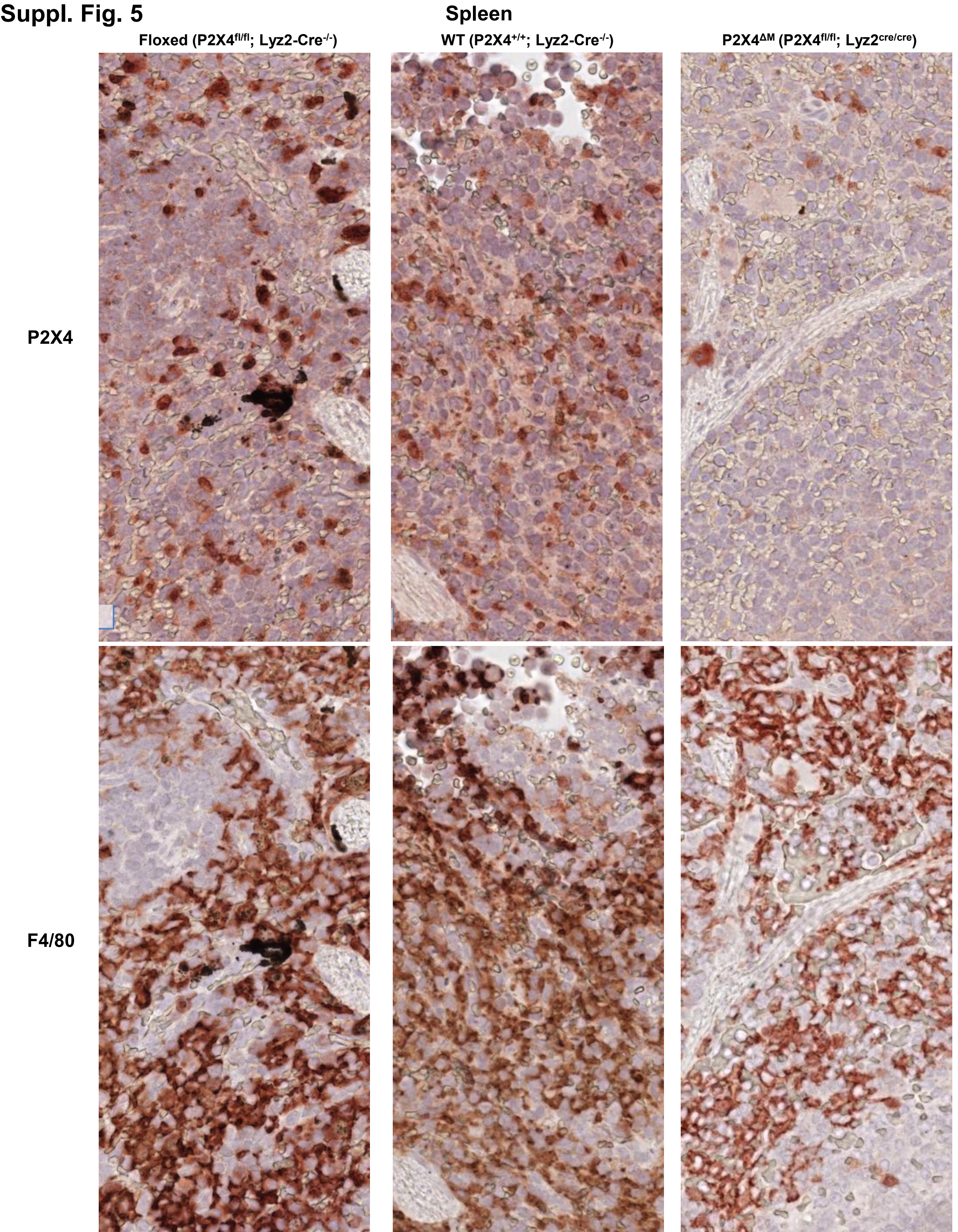
Spleen
Floxed (P2X4fl/fl; Lyz2-Cre-/-)
WT (P2X4+/+; Lyz2-Cre-/-)
P2X4ΔM (P2X4fl/fl; Lyz2cre/cre)
P2X4
F4/80

Suppl. Fig. 6

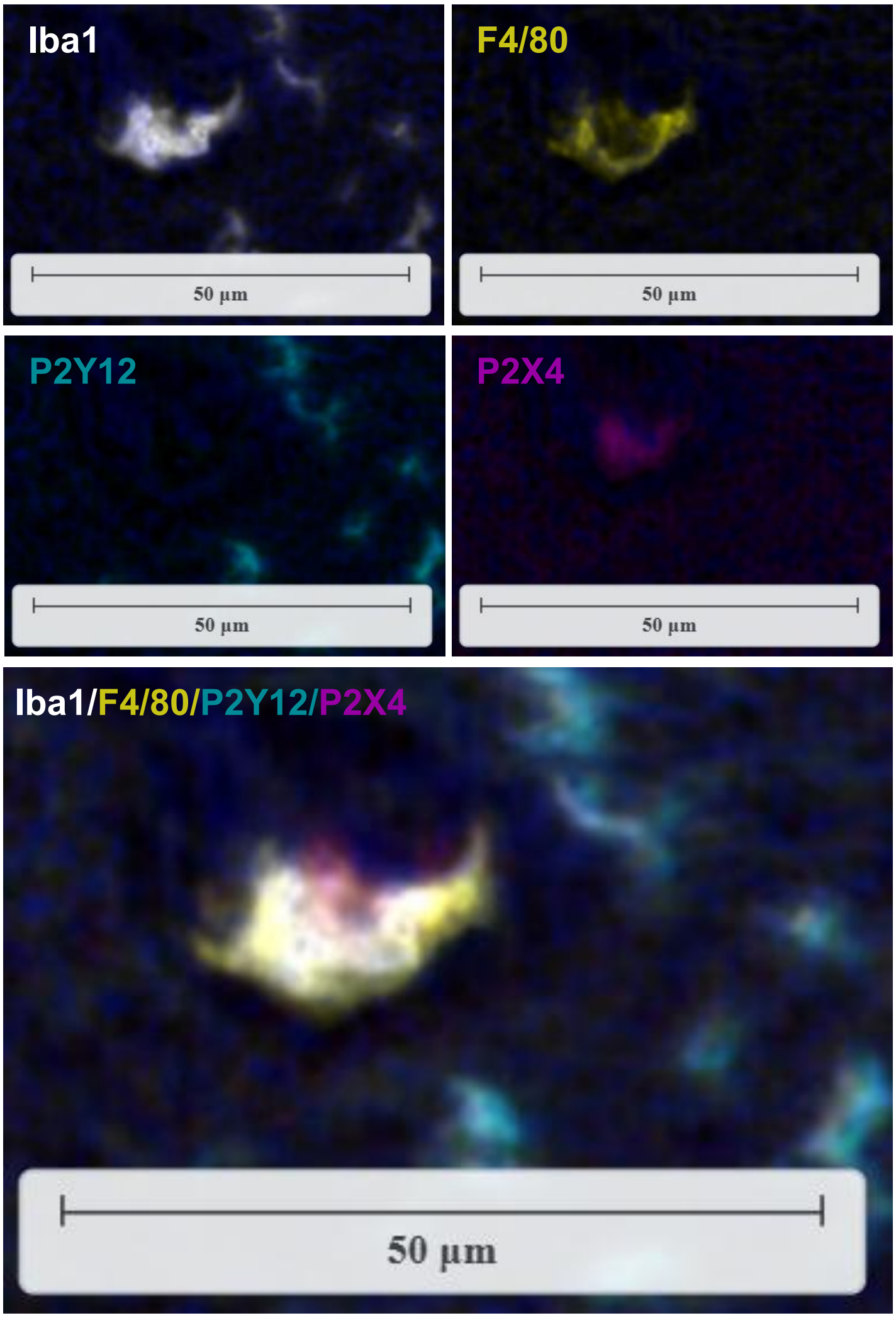
Iba1
50 µm
F4/80
50 µm
P2Y12
50 µm
P2X4
50 µm
Iba1/F4/80/P2Y12/P2X4
50 µm

Suppl. Fig. 7

Liver

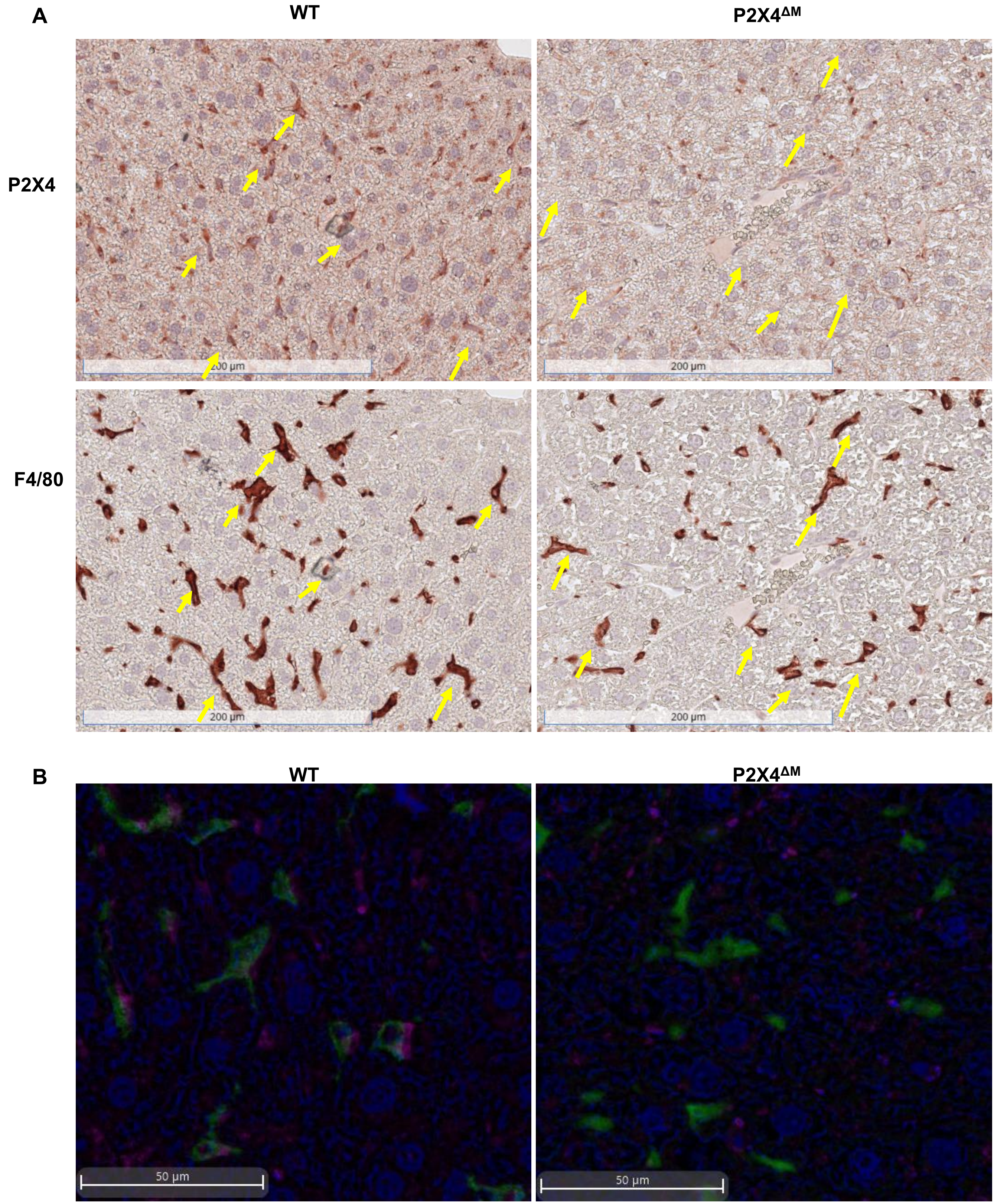

Suppl. Fig. 8

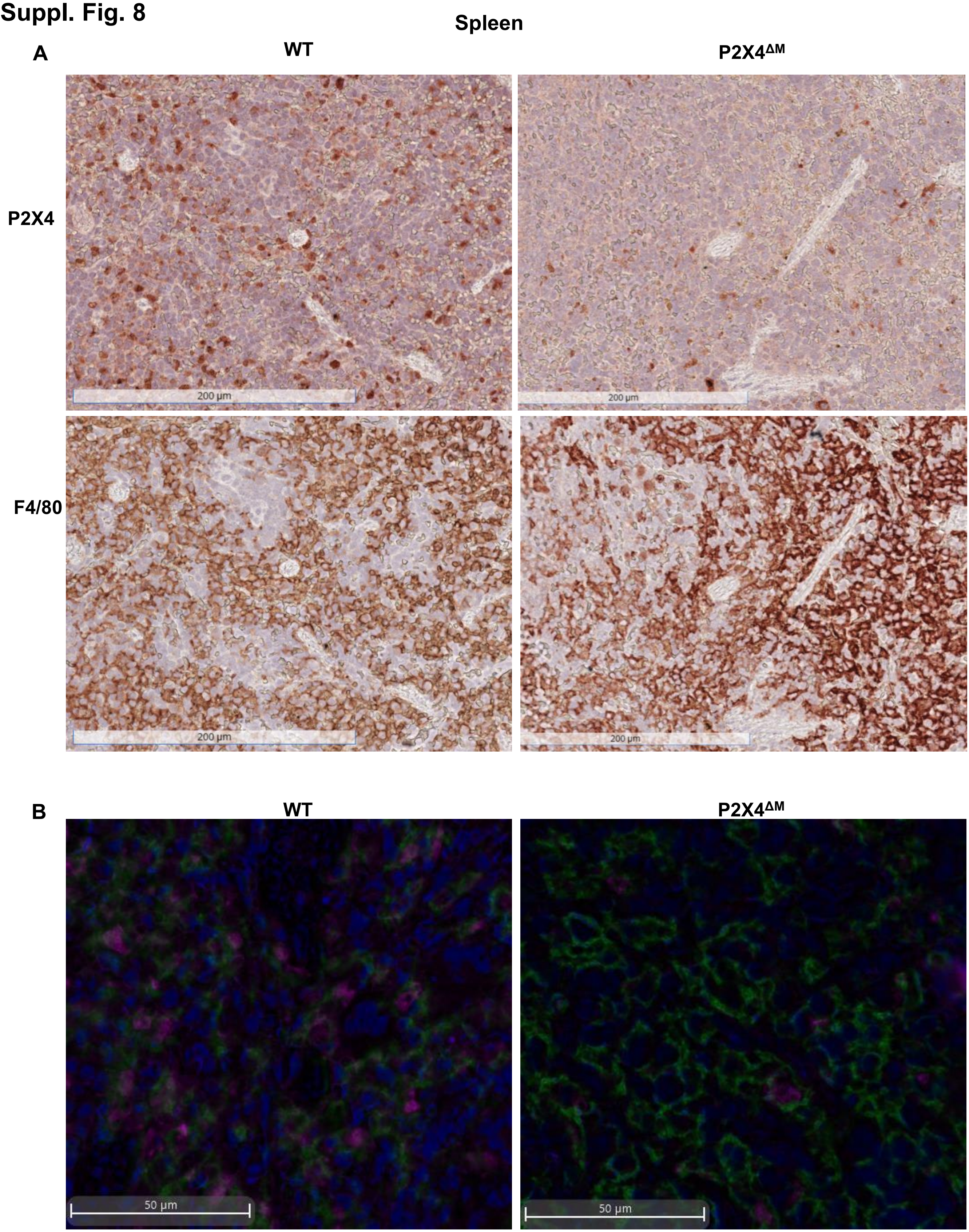
Spleen
A
WT
P2X4$^{\Delta M}$
P2X4
F4/80
200 µm
200 µm
200 µm
200 µm
B
WT
P2X4$^{\Delta M}$
50 µm
50 µm

Suppl. Fig. 9

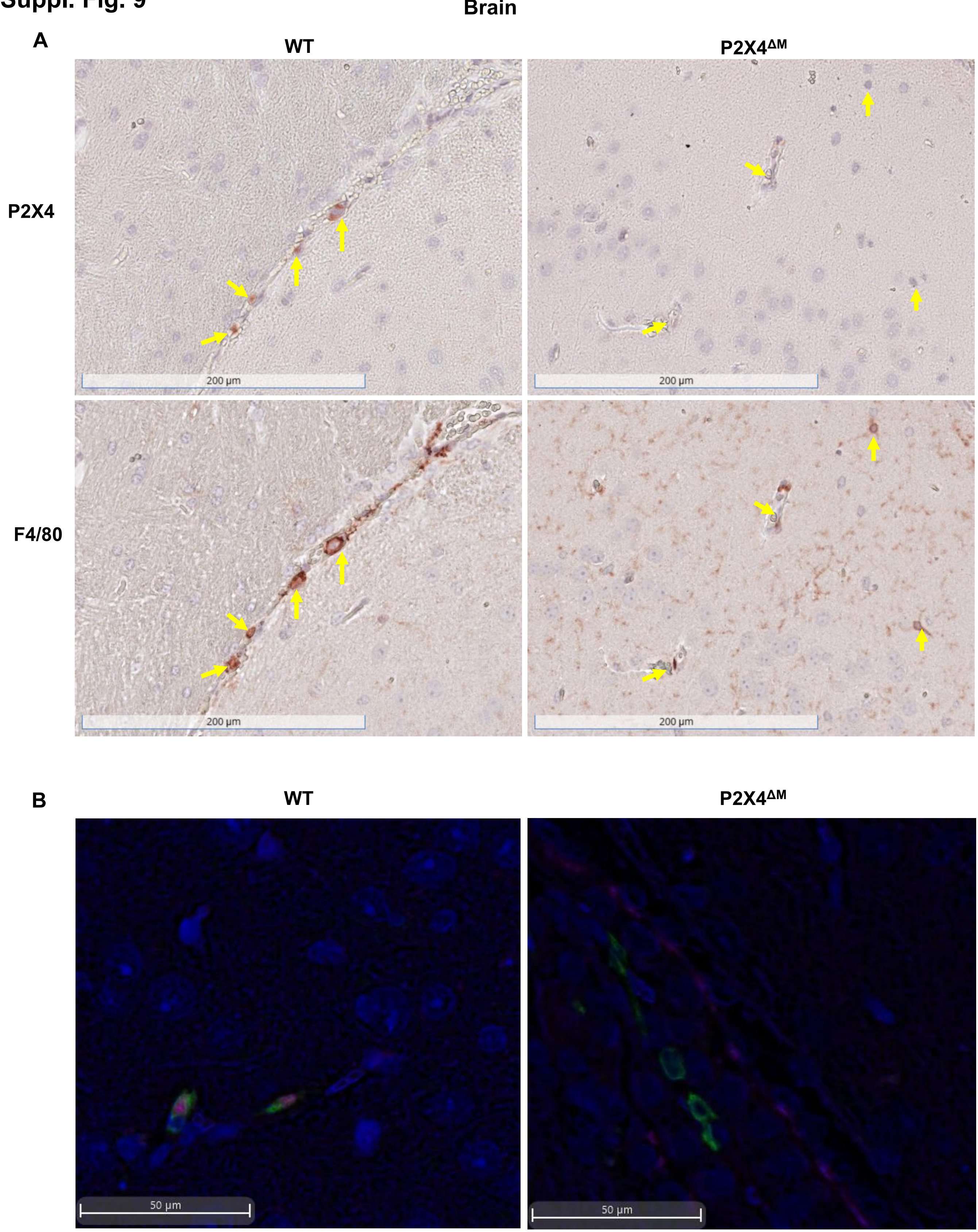

Suppl. Fig. 10

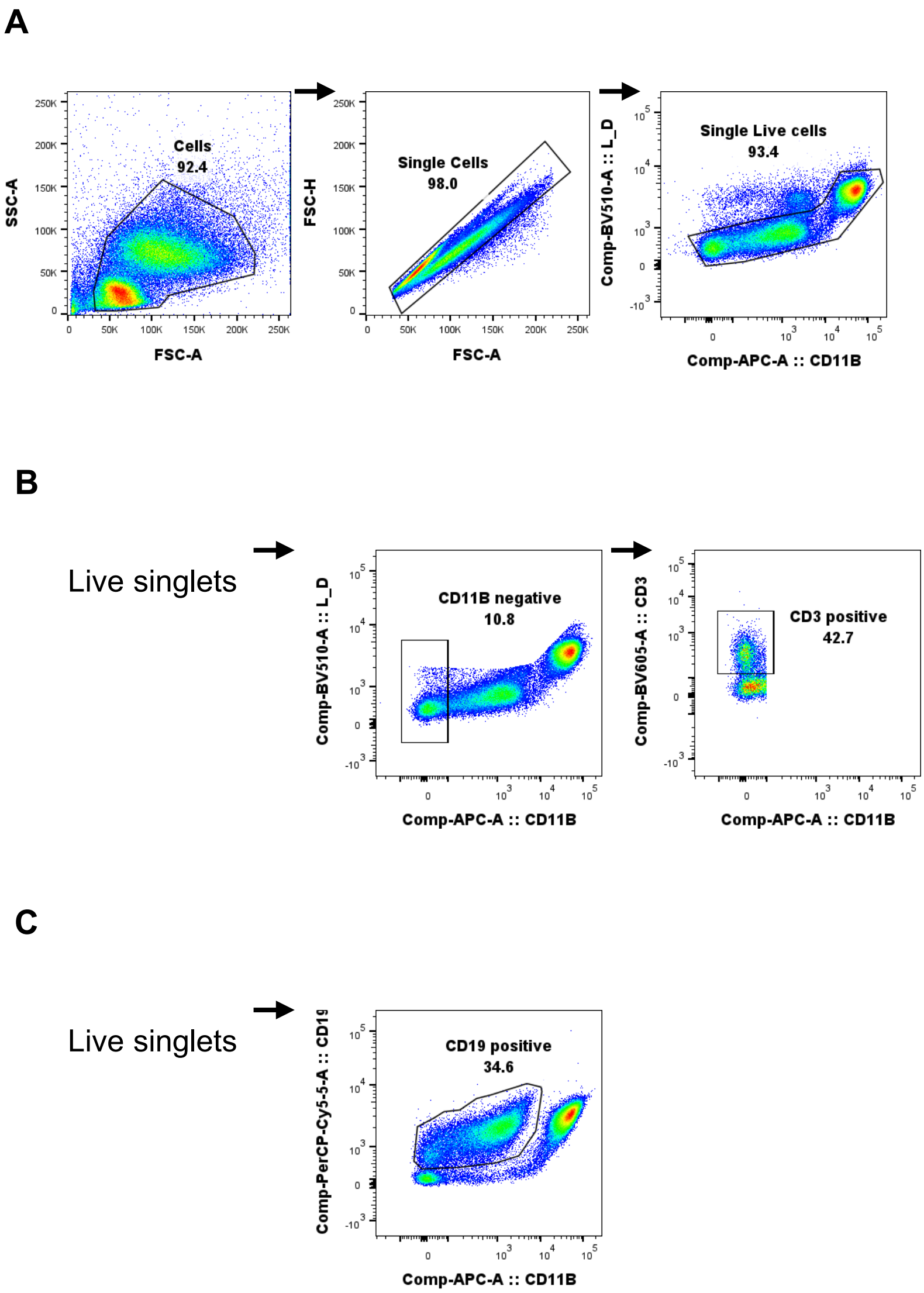
A
Cells
92.4
SSC-A
FSC-A
Single Cells
98.0
FSC-H
FSC-A
Single Live cells
93.4
Comp-BV510-A :: L_D
Comp-APC-A :: CD11B
B
Live singlets
CD11B negative
10.8
Comp-BV510-A :: L_D
Comp-APC-A :: CD11B
CD3 positive
42.7
Comp-BV605-A :: CD3
Comp-APC-A :: CD11B
C
Live singlets
CD19 positive
34.6
Comp-PerCP-Cy5-5-A :: CD19
Comp-APC-A :: CD11B

Suppl. Fig. 11

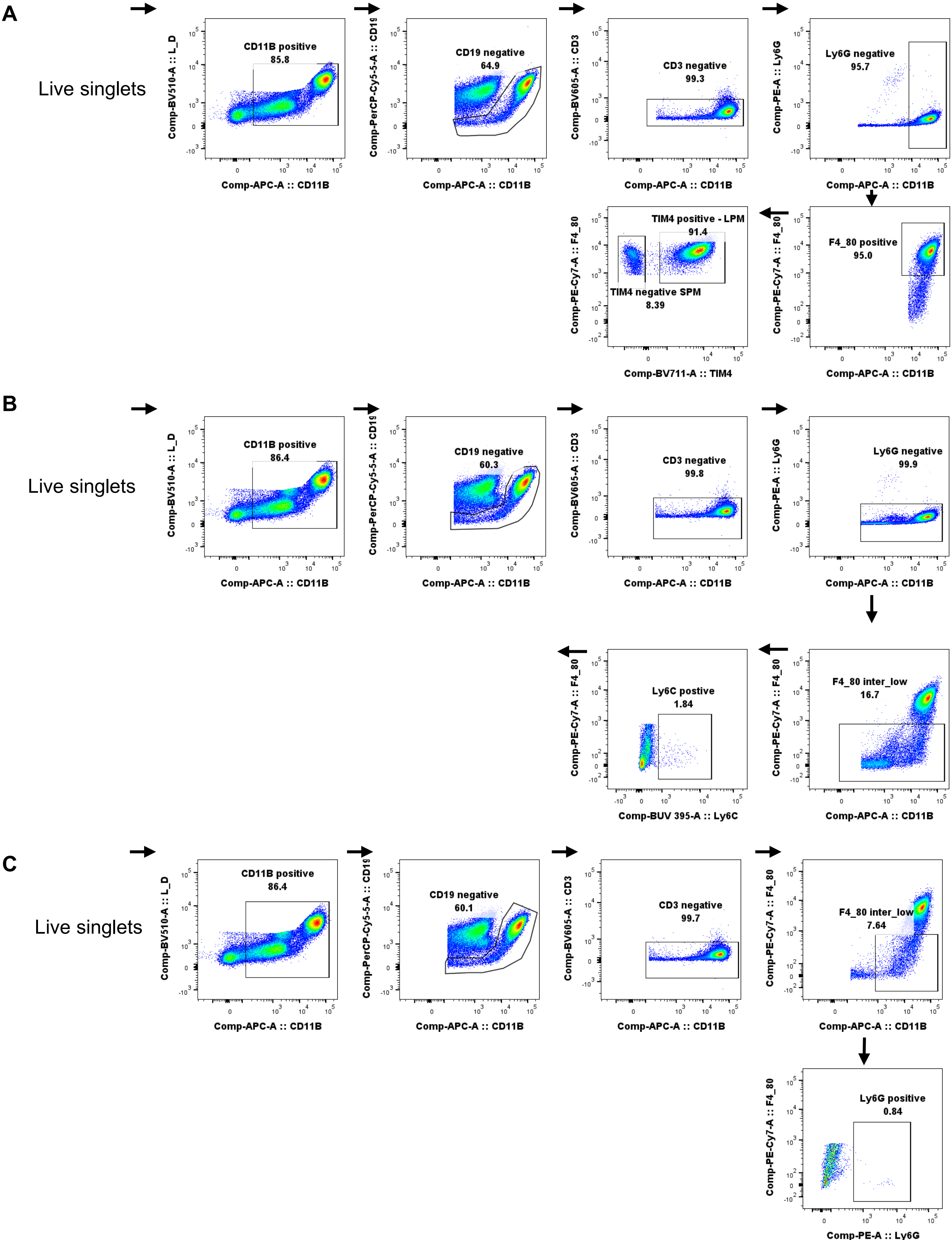
A
Live singlets
CD11B positive
85.8
Comp-BV510-A :: L_D
Comp-APC-A :: CD11B
CD19 negative
64.9
CD3 negative
99.3
Comp-BV605-A :: CD3
Ly6G negative
95.7
Comp-PE-A :: Ly6G
F4_80 positive
95.0
Comp-PE-Cy7-A :: F4_80
TIM4 positive - LPM
91.4
TIM4 negative SPM
8.39
Comp-BV711-A :: TIM4
B
Live singlets
CD11B positive
86.4
CD19 negative
60.3
CD3 negative
99.8
Ly6G negative
99.9
F4_80 inter_low
16.7
Ly6C postive
1.84
Comp-BUV 395-A :: Ly6C
C
Live singlets
CD11B positive
86.4
CD19 negative
60.1
CD3 negative
99.7
F4_80 inter_low
7.64
Ly6G positive
0.84
Comp-PE-A :: Ly6G

Suppl. Fig. 12

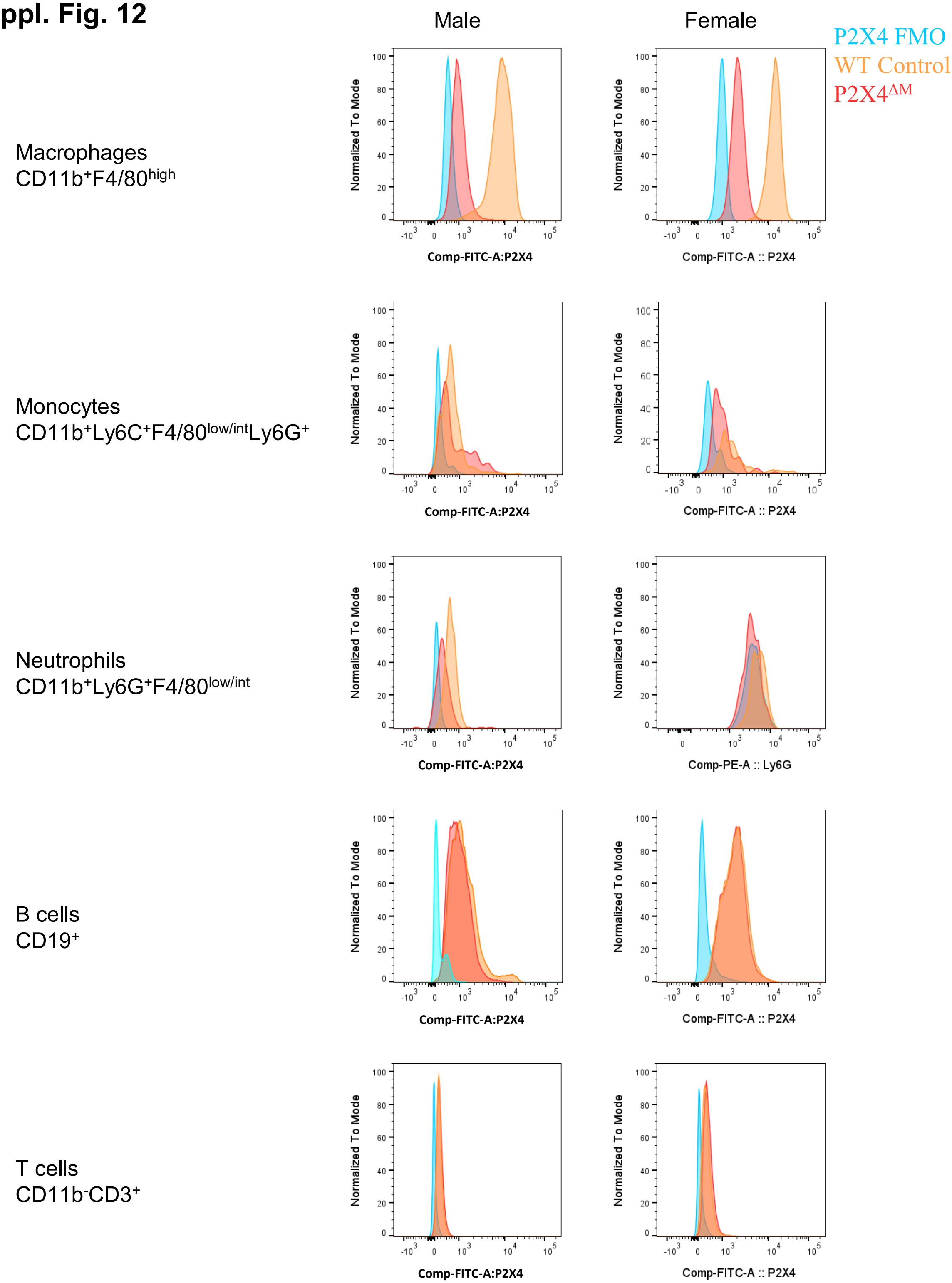
Male
Female
P2X4 FMO
WT Control
P2X4ΔM
Macrophages
CD11b+F4/80high
Monocytes
CD11b+Ly6C+F4/80low/intLy6G+
Neutrophils
CD11b+Ly6G+F4/80low/int
B cells
CD19+
T cells
CD11b-CD3+
Normalized To Mode
Comp-FITC-A:P2X4
Comp-FITC-A :: P2X4
Comp-PE-A :: Ly6G

Suppl. Fig. 13

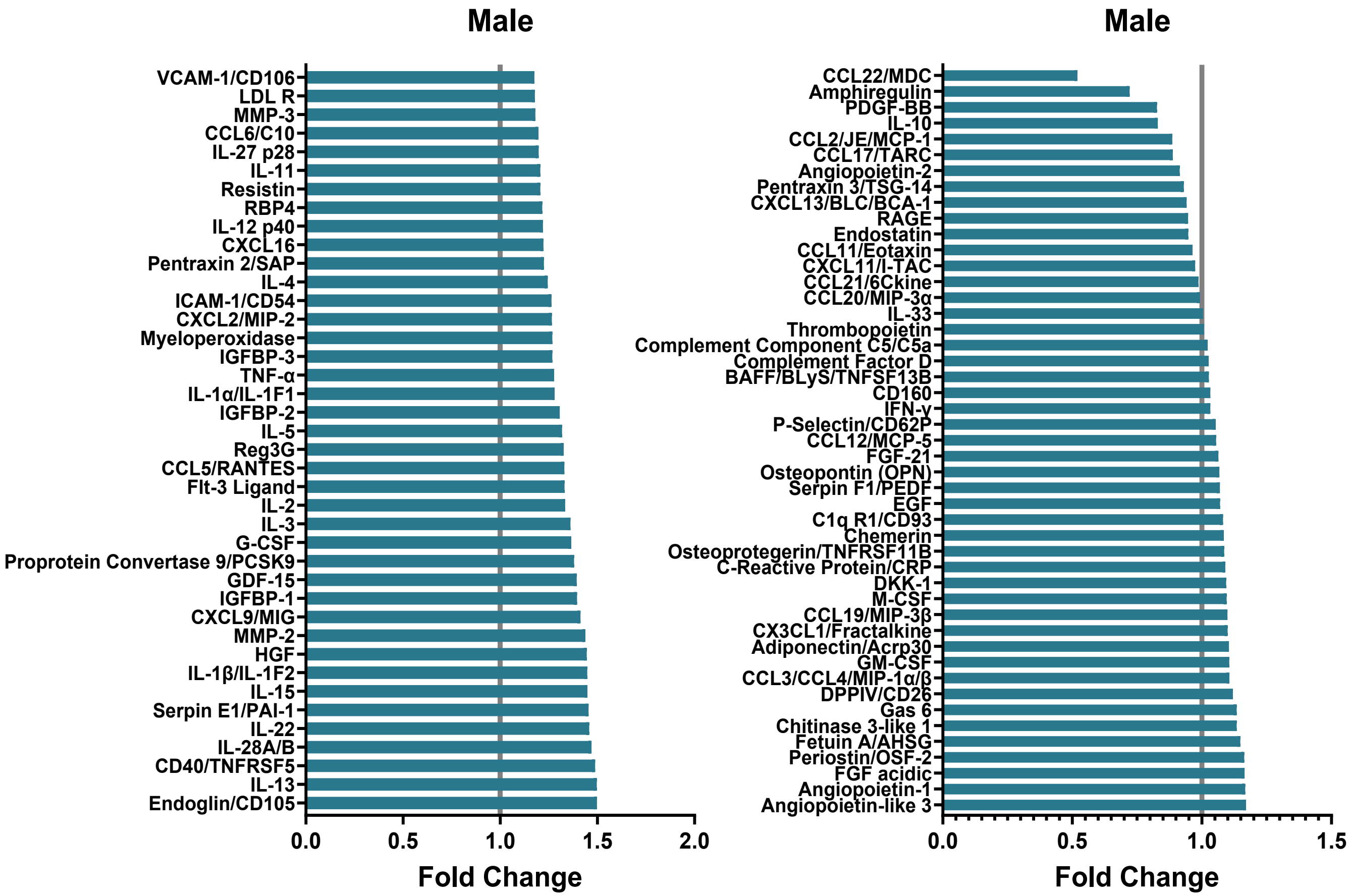
Male
VCAM-1/CD106
LDL R
MMP-3
CCL6/C10
IL-27 p28
IL-11
Resistin
RBP4
IL-12 p40
CXCL16
Pentraxin 2/SAP
IL-4
ICAM-1/CD54
CXCL2/MIP-2
Myeloperoxidase
IGFBP-3
TNF-α
IL-1α/IL-1F1
IGFBP-2
IL-5
Reg3G
CCL5/RANTES
Flt-3 Ligand
IL-2
IL-3
G-CSF
Proprotein Convertase 9/PCSK9
GDF-15
IGFBP-1
CXCL9/MIG
MMP-2
HGF
IL-1β/IL-1F2
IL-15
Serpin E1/PAI-1
IL-22
IL-28A/B
CD40/TNFRSF5
IL-13
Endoglin/CD105
0.0
0.5
1.0
1.5
2.0
Fold Change
Male
CCL22/MDC
Amphiregulin
PDGF-BB
IL-10
CCL2/JE/MCP-1
CCL17/TARC
Angiopoietin-2
Pentraxin 3/TSG-14
CXCL13/BLC/BCA-1
RAGE
Endostatin
CCL11/Eotaxin
CXCL11/I-TAC
CCL21/6Ckine
CCL20/MIP-3α
IL-33
Thrombopoietin
Complement Component C5/C5a
Complement Factor D
BAFF/BLyS/TNFSF13B
CD160
IFN-γ
P-Selectin/CD62P
CCL12/MCP-5
FGF-21
Osteopontin (OPN)
Serpin F1/PEDF
EGF
C1q R1/CD93
Chemerin
Osteoprotegerin/TNFRSF11B
C-Reactive Protein/CRP
DKK-1
M-CSF
CCL19/MIP-3β
CX3CL1/Fractalkine
Adiponectin/Acrp30
GM-CSF
CCL3/CCL4/MIP-1α/β
DPPIV/CD26
Gas 6
Chitinase 3-like 1
Fetuin A/AHSG
Periostin/OSF-2
FGF acidic
Angiopoietin-1
Angiopoietin-like 3
0.0
0.5
1.0
1.5
Fold Change

Suppl Fig. 14

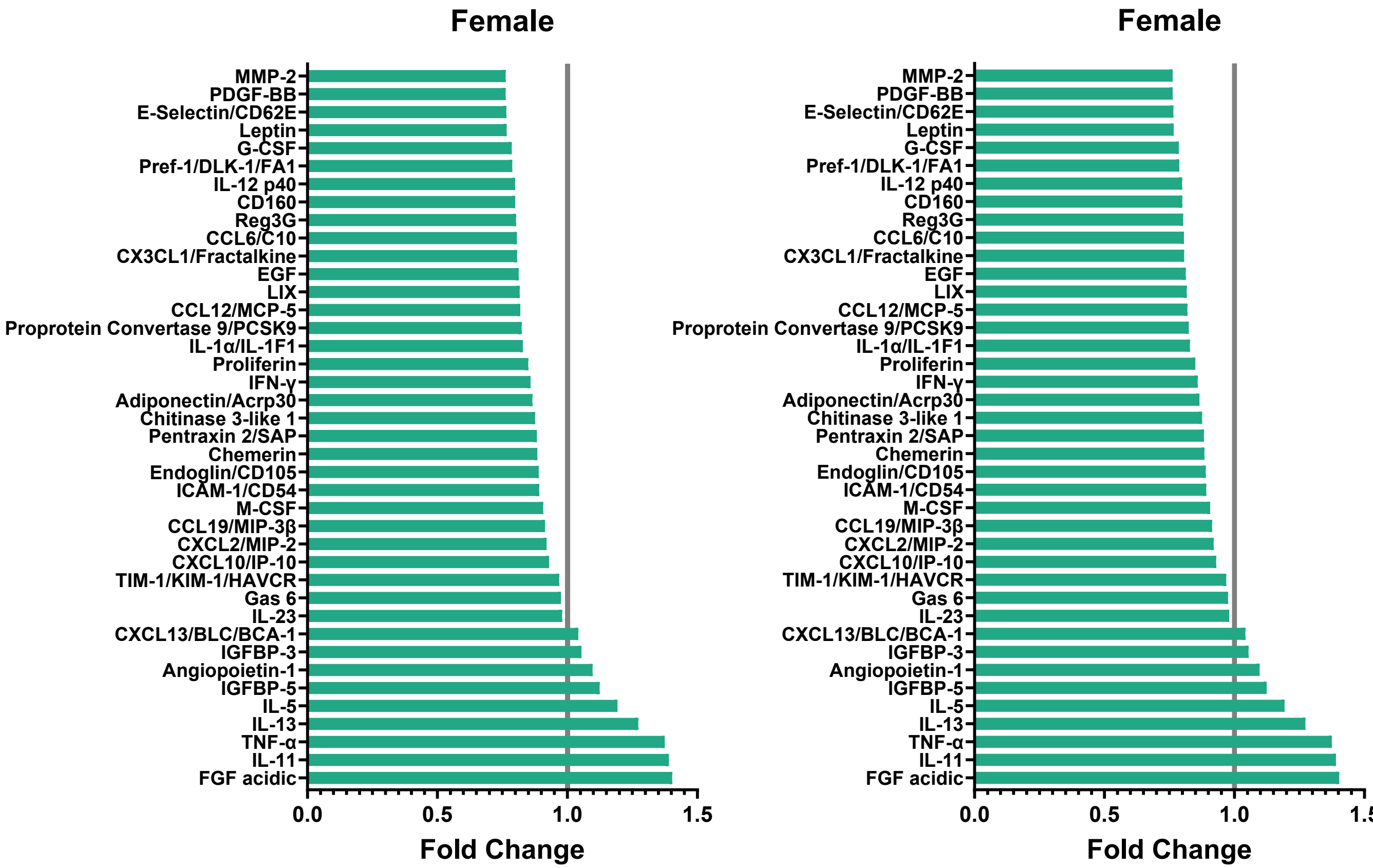
Female
MMP-2
PDGF-BB
E-Selectin/CD62E
Leptin
G-CSF
Pref-1/DLK-1/FA1
IL-12 p40
CD160
Reg3G
CCL6/C10
CX3CL1/Fractalkine
EGF
LIX
CCL12/MCP-5
Proprotein Convertase 9/PCSK9
IL-1α/IL-1F1
Proliferin
IFN-γ
Adiponectin/Acrp30
Chitinase 3-like 1
Pentraxin 2/SAP
Chemerin
Endoglin/CD105
ICAM-1/CD54
M-CSF
CCL19/MIP-3β
CXCL2/MIP-2
CXCL10/IP-10
TIM-1/KIM-1/HAVCR
Gas 6
IL-23
CXCL13/BLC/BCA-1
IGFBP-3
Angiopoietin-1
IGFBP-5
IL-5
IL-13
TNF-α
IL-11
FGF acidic
0.0
0.5
1.0
1.5
Fold Change
Female
MMP-2
PDGF-BB
E-Selectin/CD62E
Leptin
G-CSF
Pref-1/DLK-1/FA1
IL-12 p40
CD160
Reg3G
CCL6/C10
CX3CL1/Fractalkine
EGF
LIX
CCL12/MCP-5
Proprotein Convertase 9/PCSK9
IL-1α/IL-1F1
Proliferin
IFN-γ
Adiponectin/Acrp30
Chitinase 3-like 1
Pentraxin 2/SAP
Chemerin
Endoglin/CD105
ICAM-1/CD54
M-CSF
CCL19/MIP-3β
CXCL2/MIP-2
CXCL10/IP-10
TIM-1/KIM-1/HAVCR
Gas 6
IL-23
CXCL13/BLC/BCA-1
IGFBP-3
Angiopoietin-1
IGFBP-5
IL-5
IL-13
TNF-α
IL-11
FGF acidic
0.0
0.5
1.0
Fold Change
