# Supplementary material for: P2X4 purinergic receptor loss alters macrophage homeostasis, cytokine secretion, and phagocytosis in a myeloid-specific P2X4 purinergic receptor knockout mouse
Source: Front Immunol. 2026 Sep 10;17:1916582. doi: 10.3389/fimmu.2026.1916582 (PMC13600901; doi:10.3389/fimmu.2026.1916582)
Supplement: Supplementary file 2 [file DataSheet1.docx]

Supplementary Material

# 1 Supplementary Tables and Figures

## 1.1 Supplementary Tables

Supplementary Table 1. Antibody panel, fluorophores, and staining localization for flow cytometric analysis of peritoneal immune cell populations

| **Target** | **Antibody** | **Clone** | **Fluorophore** | **Staining** | **Company** | **Catalog No.** |
| --- | --- | --- | --- | --- | --- | --- |
| Myeloid cells | CD11b | M1/70 | APC | Surface | BioLegend | 101211 |
| B cells | CD19 | 6D5 | PerCP-Cy5.5 | Surface | BioLegend | 115535 |
| Pan macrophages | F4/80 | BM8 | PE-Cy7 | Surface | BioLegend | 123113 |
| LPM | TIM4 | RMT4-54 | BV711 | Surface | BD Biosciences | 745509 |
| Monocytes | Ly6C | HK1.4 | BUV395 | Surface | BD Biosciences | 128017 |
| Neutrophils | Ly6G | 1A8 | PE | Surface | BioLegend | 127607 |
| T cells | CD3 | 17A2 | BV605 | Surface | BioLegend | 100235 |
| Dead cells |  |  | Aqua |  | Thermo Fisher | L34957 |
| P2X4 purinergic receptor | P2X4* |  |  | Intracellular | Alomone Labs | APR-002 |
|  | goat anti-rabbit IgG (H+L) secondary antibody |  | CF488A |  | Biotium | 20019-1 |

***P2X4 staining was performed using a rabbit primary antibody followed by fluorophore-conjugated secondary antibody.**

Supplementary Table 2. Primer sequences and annealing temperatures used for qRT-PCR analysis

| **Gene** | **Forward Primer (5′→3′)** | **Reverse Primer (5′→3′)** | **Annealing Temperature (°C)** |
| --- | --- | --- | --- |
| Loxl2 | CAAAGAGTCGACCCTGGCAT | CGTCTTCAGTGTGCTTGCAG | 68 |
| Plxnd1 | CGTCTCCAAAGAAGGCAGGTCT | CGTTGAGGTCACTGCCGTGAAT | 60 |
| Chi3l1 | TGAACTATGCCGTGCAGTACA | AGAAGATGCCAGAGTGAAGCTC | 65 |
| Col18a1 | TACTACCTGGGCCTCCCTCAG | ATAGAATCTACCCGGGCGTC | 66 |
| Slit2 | GGCAGACACTGTCCCTATCG | ATCCAGTCTCTCGGTGTTGC | 66 |
| P2rx4 | AAAGGGCTACCAGGAACCG | CTGGGACCACATAGTCGGC | 64 |
| P2rx7 | ACATGTGGAAAGCCGGACG | TCAATGCACACAGTGGCCA | 67 |
| P2ry2 | GGCAGAGACTTGTCCGCTTT | TCCGTCTTGAGTCGCTACTG | 67 |
| Gapdh | CATCACTGCCACCCAGAAGACTG | ATGCCAGTGAGCTTCCCGTTCAG | 60 |

## 1.2 Supplementary Figure captions

**Supplementary Figure 1**. Optimization and validation of the multiplex immunohistochemistry panel for P2X4 purinergic receptor staining in A) mouse liver and spleen and B) brain tissues. Representative 40× images demonstrate effective removal of primary antibodies between sequential staining rounds, confirming specificity of multiplex staining and absence of signal carryover between staining cycles.

**Supplementary Figure 2.** Workflow for HALO dual-marker quantification. Representative images of P2X4 (pink) and F4/80 (green) following tissue registration in HALO. The corresponding HALO analysis output were used to quantify the percentage of overlapping P2X4/F4/80 positive area within annotated regions of interest.

**Supplementary figure 3.** Schematic representation of the A) breeding strategy used to generate P2X4^ΔM^ mice. B) Body weights of female and male WT and P2X4^ΔM^ mice measured over time. Solid lines indicate group means, and dotted lines represent individual mice for each genotype. C) Bar graph showing the total number of pups produced per cage from WT and P2X4^ΔM^ breeding pairs over a 3-month period. Each dot represents one cage; bars indicate mean ± SD. D) Scatter plot showing the number of pups per litter from WT and P2X4^ΔM^ breeding pairs. Each dot represents one litter, and horizontal lines indicate the mean litter size for each genotype. Statistical significance was determined using two-way ANOVA or Welch’s t test as appropriate (*P < 0.05, **P < 0.01). Created in BioRender. Maynard, J. (2026) [https://BioRender.com/8c09d5k](https://biorender.com/8c09d5k)

**Supplementary figure 4.** Representative multiplex IHC images showing P2X4 purinergic receptor protein expression in F4/80+ Kupffer cells from Floxed, WT and P2X4^ΔM^ mice.

**Supplementary figure 5.** Representative multiplex IHC images showing P2X4 purinergic receptor protein expression in splenic red pulp macrophages from Floxed, WT and P2X4ΔM mice.

**Supplementary Figure 6**. HALO analysis showing P2X4 purinergic receptor expression in Iba1⁺F4/80⁺ cells, but not in Iba1⁺P2Y12⁺ cells, in mouse brain tissue. Representative images showing Iba1 (white), F4/80 (yellow), P2Y12 (cyan), and P2X4 purinergic receptor (pink) staining in brain sections from naive mice. The merged image demonstrates robust P2X4 purinergic receptor expression in an Iba1⁺F4/80⁺ cell population with no detectable P2Y12 signal. Scale bar = 50 µm.

**Supplementary figure 7.** Representative multiplex IHC images showing loss of P2X4 purinergic receptor protein expression in F4/80+ Kupffer cells from P2X4^ΔM^ mice. Yellow arrows indicate F4/80^+^ macrophages. Scale bar = 200 µm. (B) HALO fused images showing P2X4 purinergic receptor (pink) and F4/80 (green) expression in mouse liver tissue. Scale bar = 50 µm.

**Supplementary figure 8.** Representative multiplex IHC images showing loss of P2X4 purinergic receptor protein expression in F4/80^+^ splenic red pulp macrophages from P2X4^ΔM^ mice.  Scale bar = 200 µm. (B) HALO fused images showing P2X4 purinergic receptor (pink) and F4/80 (green) expression in mouse spleen tissue. Scale bar = 50 µm.

**Supplementary figure 9.** Representative multiplex IHC images showing loss of P2X4 purinergic receptor protein expression in F4/80^+^ macrophages in brain tissues from P2X4^ΔM^ mice. Yellow arrows indicate F4/80^+^ macrophages. Scale bar = 200 µm. (B) HALO fused images showing P2X4 purinergic receptor (pink) and F4/80 (green) expression in mouse brain tissue. Scale bar = 50 µm.

**Supplementary figure 10**. Flow cytometry gating strategy for live cells and lymphocyte populations from peritoneal lavage. A) Cells were first gated on total cells based on FSC-A versus SSC-A, followed by exclusion of doublets using FSC-A versus FSC-H to obtain singlets. Live cells were then selected prior to downstream gating. B) T cell gating: From live singlets, myeloid cells were excluded by gating out CD11b^+^ populations. T cells were then identified as CD3^+^ cells within the CD11b^−^ fraction. C) B cell gating: From live singlets, B cells were identified as CD19^+^ cells. All plots shown are representative of the gating strategy applied consistently across samples.

**Supplementary Figure 11**. Flow cytometry gating strategy for peritoneal myeloid cell populations. A) Myeloid/macrophage gating: Cells were first gated on live singlets, followed by selection of CD11b^+^ myeloid cells. B cells, T cells and neutrophils were excluded by gating out CD19^+^, CD3^+^, Ly6G^+^ populations, respectively. Within the CD11b^+^CD19^−^CD3^−^ compartment, macrophages were identified based on F4/80 expression. LPMs and SPMs were further distinguished based on differential expression of TIM4. B). Neutrophil gating: Live singlets and CD11b^+^CD19^−^CD3^−^ cells, with Ly6G^+^F4/80^low/−^ were identified as neutrophils. C) Monocyte gating: Live singlets and CD11b^+^CD19^−^CD3^−^ cells, with Ly6C^+^Ly6G^−^ cells were defined as monocytes. All plots shown are representative of the gating applied across samples.

**Supplementary Figure 12**. Representative flow cytometry histograms showing P2X4 expression (FITC) in macrophages, monocytes, neutrophils, B cells, and T cells from male and female mice. Fluorescence minus one (FMO) controls (blue) were used to define background fluorescence and gating thresholds. WT control samples (orange), P2X4^ΔM^ mice (red). Histograms are representative of samples analyzed from each group, with expression plotted as fluorescence intensity (Comp-FITC-A) versus cell count.

**Supplementary Figure 13.** Complete Cytokine Array dataset showing fold changes in serum cytokine and growth factor levels in male P2X4^ΔM^ mice relative to WT

**Supplementary Figure 14.** Complete Cytokine Array dataset showing fold changes in serum cytokine and growth factor levels in female P2X4^ΔM^ mice relative to WT
